# Less is more: miniaturized heme enzymes with broad reactivity

**DOI:** 10.1039/d6sc05172c

**Published:** 2026-09-16

**Authors:** Linda Leone, Daniele D'Alonzo, Roberta Russo, Alessandra Esposito, Angela Lombardi

**Affiliations:** a Department of Chemical Sciences, University of Napoli Federico II Napoli Italy linda.leone@unina.it alombard@unina.it

## Abstract

Heme enzymes are among the most efficient and versatile biocatalysts known, exploiting the rich redox chemistry of iron porphyrins to perform a wide range of transformations. Replicating and expanding this catalytic diversity within artificial systems remains a major challenge in bioinorganic chemistry. Among the strategies developed to design and engineer artificial heme enzymes, miniaturization occupies a distinctive position. Rather than reproducing the complexity of natural protein scaffolds, it builds on the structural knowledge of natural enzymes and works backward to identify the minimal peptide fragment that retains the essential information required for metal cofactor binding and catalytic function. Mimochromes (MCs) are among the most illustrative examples of this approach: a compact, synthetically accessible peptide scaffold surrounds a metalloporphyrin cofactor, providing a well-defined yet adaptable active site. This perspective outlines the design and development of MCs, from the early prototypes to the current benchmark catalyst MC6*a. Attention is devoted to the role of the MC scaffold in accommodating a broad range of substrates and modulating the reactivity of different metal ions. These features have enabled the development of first-in-class catalysts among both natural enzymes and artificial analogues, capable of promoting a remarkable variety of highly efficient and selective catalytic transformations spanning peroxidation and peroxygenation chemistry, electro- and photocatalytic hydrogen evolution, and electrocatalytic CO_2_ reduction. After discussing the catalytic features of MCs in comparison with selected natural enzymes and small-molecule catalysts, we identify key future directions for this research area. In this context, the integration of MCs into functional nanomaterials highlights emerging opportunities for the development of hybrid catalytic platforms. Future efforts will also focus on broadening the scope of accessible transformations, unlocking their potential in chemical synthesis, diagnostics, energy conversion, and environmental remediation.

## Abstract 2

Less is more: inspired by natural heme proteins, the miniaturized enzyme MC6*a shows how a small peptide scaffold can deliver remarkable catalytic versatility, driving reactions spanning from selective oxidations to light-driven hydrogen production.

## Graphical abstract

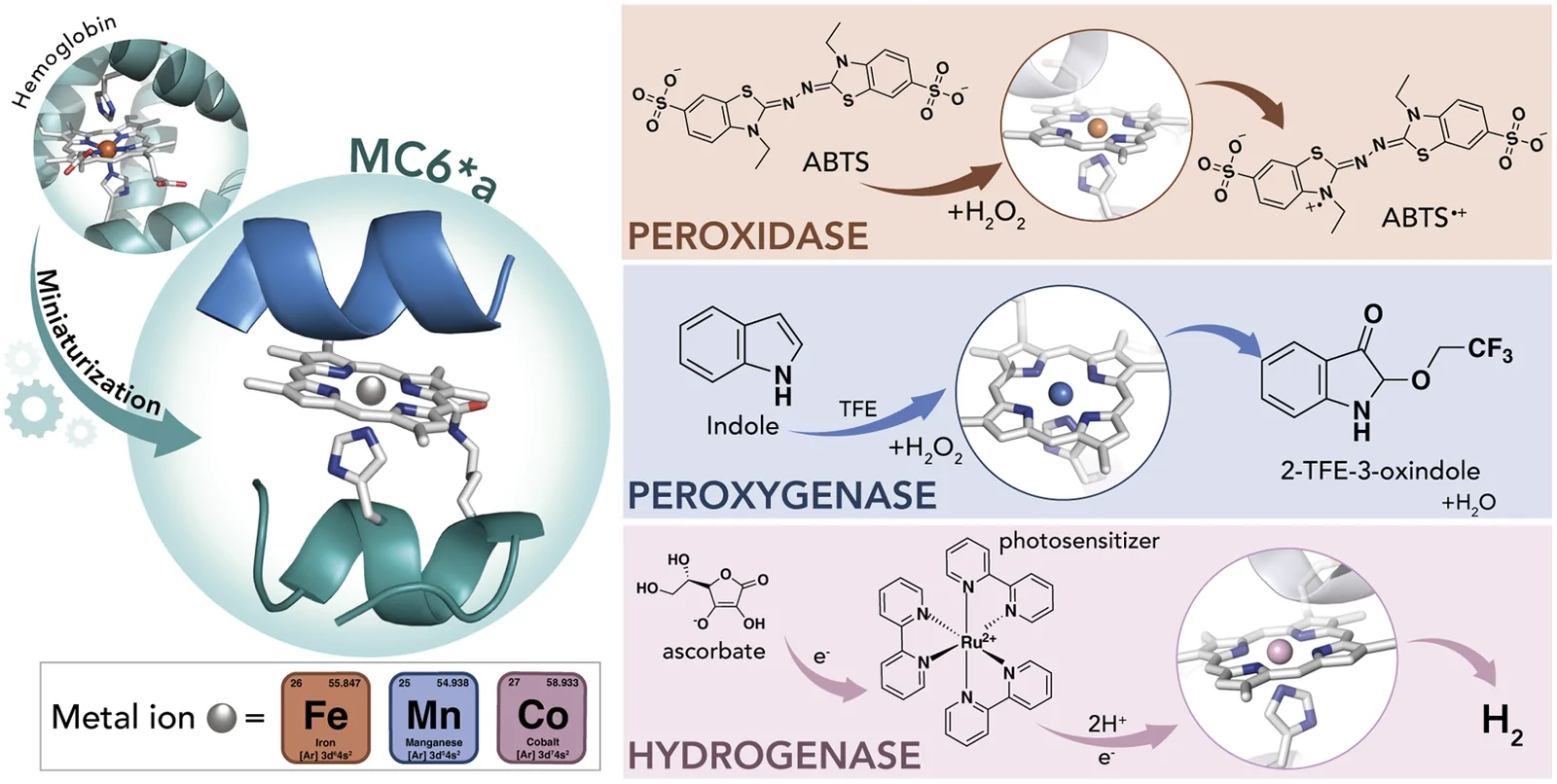

## Introduction

Understanding the structural features that govern the catalytic functions of metalloenzymes and reproducing them in artificial systems has been a major focus in bioinorganic chemistry over the past decades.^1–3^ Considerable efforts have been devoted to deciphering the intricate network of interactions between metal cofactors and the surrounding protein matrix,^1,4^ which directs the unique reactivity of the cofactor by controlling key factors such as the metal reduction potential, substrate and product selectivity, and the stabilization of highly reactive species.^1,2^ In this context, our group has developed a variety of metalloproteins that bind heme,^5^ iron–sulfur,^6^ diiron^7^ or dicopper^8^ centers, as well as a type 2 copper center through the histidine-brace motif.^9^ This perspective focuses on artificial heme enzymes. Indeed, heme is the most widespread prosthetic group among metalloproteins, displaying unparalleled versatility by leveraging the rich redox chemistry of iron coupled with the non-innocent redox behavior of the porphyrin ligand.^10,11^ While the functional diversity of heme enzymes is strongly influenced by the nature of the proximal axial ligand,^4,5^ even proteins sharing identical first coordination shell can perform very different functions, ranging from oxygen transport to small-molecule activation.^1^ Building on structural and mechanistic insights, researchers have developed a variety of strategies to reproduce, reshape or repurpose the steric and electronic environment of the heme cofactor in artificial enzymes.^1,5,12–15^ Through computational design,^16^ chemical^14^ and biochemical methods,^1^ as well as protein engineering techniques,^12^ it is possible to tailor both first and second coordination shell interactions to create new functional metal-binding sites, incorporating either natural hemes or synthetic metalloporphyrins.^15,17^

**Fig. 1:**
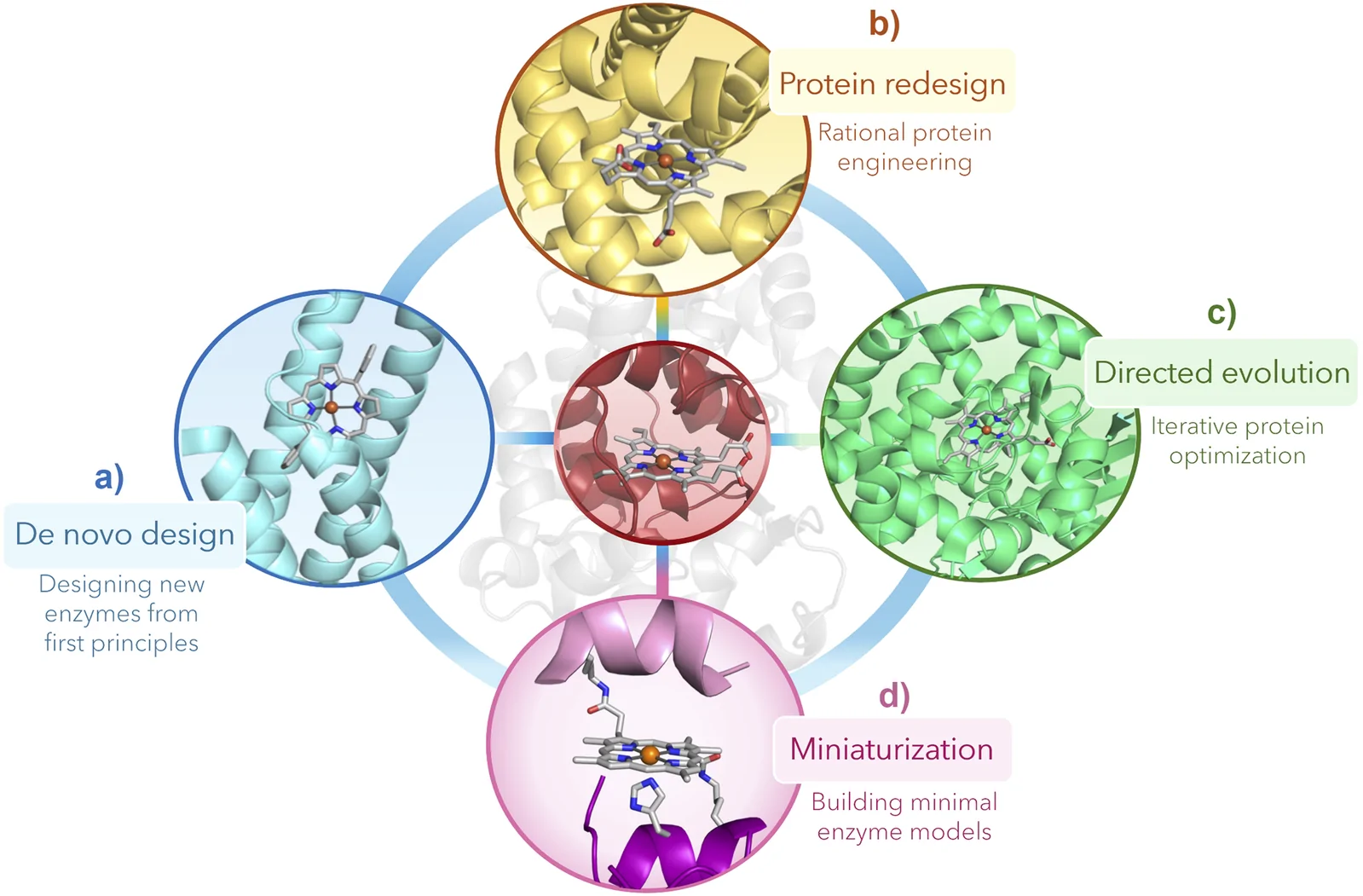
Strategies for the design and engineering of artificial heme enzymes. (a) *De novo* design (light blue circle: catalytic heme binding four-helix bundle acting as carbene transferase (Novochrome), designed by DeGrado and coworkers,^32^ PDB ID: 9CTE). (b) Protein redesign (yellow circle: modified myoglobin as biosynthetic model of heme-copper oxidase, developed by Lu and coworkers,^57^ PDB ID: 7KYR. (c) Directed evolution (green circle: cytochrome P411 as C–H amination catalyst, engineered in the Arnold lab,^64^ PDB ID: 5UCW). (d) Miniaturization (pink circle, metal mimochrome VI*a (M-MC6*a) as multipurpose biocatalyst, designed by Lombardi and coworkers.^5^ Red circle: close-up view of the active site of horseradish peroxidase (HRP), isozyme C (PDB ID: 1HCH).

The construction of artificial heme enzymes currently relies on four main complementary strategies (Fig. 1). In the late 1980s, DeGrado and coworkers pioneered the *de novo* protein design^18,19^ (Fig. 1a), *i.e.* the design of proteins from scratch, which has enabled the generation of folded and functional proteins, including heme enzymes.^2^ An improved understanding of the principles governing protein–metal interactions,^19^ together with rapid advances in machine-learning-based structure prediction tools,^20^ have further facilitated the *de novo* design of metalloporphyrin-containing enzymes for a variety of applications. A major breakthrough came with the development of the Rosetta platform by Baker and co-workers.^21^ It has transformed the *de novo* protein design by enabling the routine generation of proteins with entirely new folds and functions beyond those found in nature,^22^ while also providing a versatile framework for modelling and designing protein–protein and protein–ligand interactions.^23^ These capabilities have greatly expanded the ability to engineer proteins that selectively recognize and tightly bind cofactors and substrates.^24,25^

Among *de novo* designed scaffolds, α-helical bundles, and especially four-helix bundles, are the most widely employed, owing to their high stability and tolerance to multiple mutations.^26^ While initial designs focused on oxygen binding^27^ and electron transfer,^28^ the functional repertoire of four-helix bundle heme proteins has expanded considerably over time. Notable examples include c-type heme maquettes (CTMs),^27^ which are able to display both peroxidase^29^ and carbene transferase^30^ activities. Four-helix bundle proteins have also been designed to host abiological metalloporphyrin cofactors,^31–33^ displaying peroxygenase^31,34^ and enantioselective carbene transferase^32,33^ activities or allosterically modulating the phenol oxidase activity of a diiron center in a two-domain protein.^35^ More recently, the *de novo* metalloprotein MPP1 was shown to stabilize a Mn^V^-nitrido species and mediate nitrogen-atom transfer chemistry, further expanding the functional repertoire accessible to helical bundle proteins.^36^ Other α-helical-based designs, including coiled coils and solenoid repeats, provide tunable systems with switchable or adaptable catalytic functions.^14,25,37,38^ Although heme is often found within α-helical structures in natural proteins, *de novo* heme-binding β-sheet miniproteins^39,40^ and self-assembling amyloid peptides^41–43^ have expanded the structural diversity of artificial heme proteins, forming stable catalytic systems with diverse activities, including peroxidase^39,42^ and carbene transferase.^43^

Protein redesign (Fig. 1b) introduces rational modifications into natural metalloproteins, with the aim of introducing, enhancing or altering their catalytic functions.^1^ These modifications include point mutations,^44^ active-site remodelling^45^ or metal cofactor replacement.^15^ This approach benefits from a pre-existing, stable, and well-characterised protein architecture, enabling the designer to identify and modify key features while preserving the global structure. In this context, reconstitution of myoglobin (Mb) with synthetic metalloporphyrinoids, pioneered by Hayashi and coworkers^46–48^ has unlocked challenging transformations including cyclopropanation,^46^ methane generation,^48^ CO_2_ reduction,^49^ aldoxime dehydration,^50^ and reductive dearomatization.^51^ Further, Hartwig and coworkers showed that reconstitution of Mb or P450s with an iridium porphyrin cofactor was a valuable strategy for the development of selective carbene or nitrene transferases.^52,53^ Moreover, site-directed mutagenesis has enabled systematic tuning of the heme reduction potential through second-shell and axial ligand modifications, with either canonical^54^ or synthetic amino acids,^55,56^ demonstrating how subtle changes can translate into dramatically different catalytic outcomes. Site-directed mutagenesis has also been employed by Lu and coworkers to install entirely new metal-binding sites adjacent to an existing cofactor.^45^ This generated catalytic models of heteronuclear metalloenzymes, such as heme-copper oxidases,^57^ nitric oxide reductases^58^ and sulfite reductases.^59^ Beyond modifications of natural heme proteins, the incorporation of synthetic metalloporphyrins into non-heme protein scaffolds has emerged as a powerful design strategy. A notable example is the supramolecular anchoring of synthetic cofactors to streptavidin (Sav) through the biotin–Sav technology.^60^ This approach has recently enabled the development of genetically optimized artificial peroxidases, whose activity rivals that of natural enzymes. This work further demonstrates how targeted engineering of second-shell residues can substantially enhance catalytic performance of artificial metalloenzymes.^60^

In contrast to the structure-based design strategies described above, directed evolution (Fig. 1c) emulates Darwinian selection in the laboratory by iteratively introducing random or semi-rational mutations to a protein sequence and screening the resulting variant libraries for enhanced or novel functions.^61,62^ Unlike rational design, directed evolution does not require prior mechanistic knowledge of the desired transformation, making it particularly powerful when the target activity has no natural counterpart. Applied to heme enzymes, this approach, pioneered by Arnold,^61,62^ Reetz^63^ and coworkers, has generated some of the most striking examples of functional expansion reported to date, opening new avenues in biocatalysis.^61–63^ Successive rounds of mutation and selection applied to cytochrome P450s,^12,64^ Mb^13^ and other heme proteins^65^ have radically reprogrammed their reactivity, enabling highly selective transformations unprecedented in natural systems,^61,66^ including the formation of C–C, C–Si, C–B, C–N, and C–S bonds,^12,67^ as well as metal-hydride hydrogen atom transfer (MHAT).^68^ Although directed evolution relies on an intrinsically combinatorial strategy, it should not be viewed as an alternative to structure-based design, but rather as a complementary approach. Indeed, directed evolution has been successfully employed to optimize the activity of several recently developed *de novo* heme proteins. This synergy has been most powerfully exemplified by the designed heme-binding four-helix bundles called NovoChromes,^32^ which have been evolved to achieve exceptional carbene transferase activity. Through the combination of *de novo* design and directed evolution, these biocatalysts have also been engineered to promote the unprecedented Ge–H insertion reaction.^33^

**Fig. 2:**
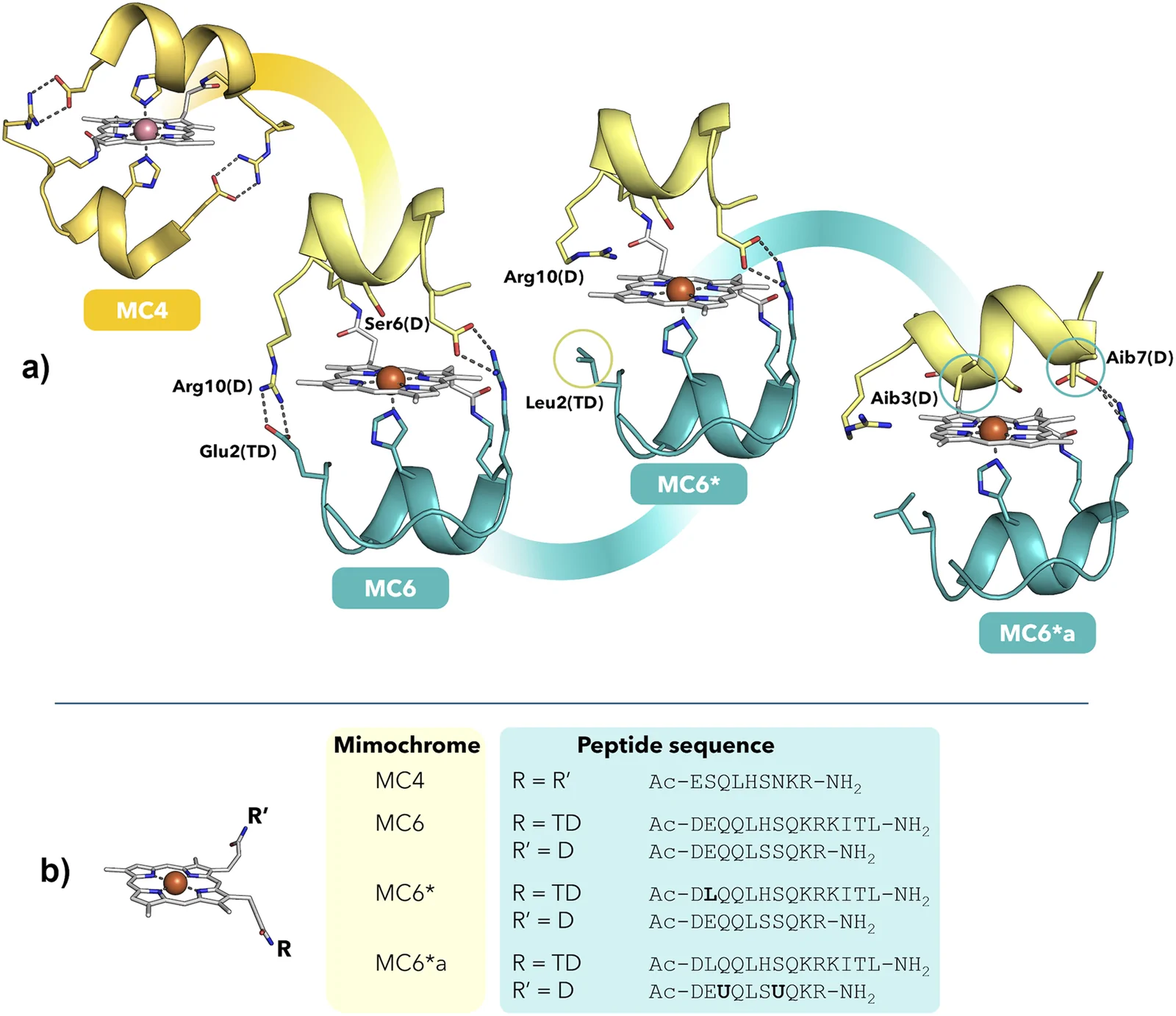
Optimization of the MC scaffold. Starting from the six-coordinate model MC4 and progressing to the five-coordinate MC6, characterized by peroxidase activity, the scaffold was further optimized to generate the improved analogues MC6* and MC6*a. Panel (a) shows the NMR structure of MC4 (in its cobalt complex, PDB ID: 1VL3), together with the designed models of MC6, MC6* and MC6*a. In these latter, tetradecapeptide (TD) and decapeptide (D) chains are depicted in teal and yellow, respectively. Mutations introduced in each round of MC6 redesign are highlighted in circles. Panel (b) shows the sequences of peptide chains (R and R′) linked to DPIX in the represented mimochromes. Mutations among MC6 analogues are reported in bold. U indicates aminoisobutyric acid (Aib).

Among the various design strategies, miniaturization (Fig. 1d) occupies a distinctive position. While it shares with *de novo* design the aim of engineering catalytic sites within artificial protein or peptide scaffolds, it places a strong emphasis on minimizing structural complexity. Indeed, *de novo* protein design has progressively evolved toward the construction of medium-to large-sized protein architectures that increasingly recapitulate the structural and functional complexity of natural systems.^2^ Miniaturization, by contrast, follows the opposite direction: rather than building complexity from scratch, it builds on the structural knowledge of natural metalloenzymes and works backward to identify the minimal peptide framework capable of reproducing key features of the native active site, including geometry, metal-binding, and catalytic activity.^4,5,69^ Minimalist models of heme enzymes are well represented in the literature. In many cases, natural or synthetic metalloporphyrins have been functionalized with chemical moieties of varying complexity, which assist catalysis by promoting substrate activation or modulating the reactivity of the metal center.^70,71^ However, the absence of a proper peptide scaffold, essential for conferring selectivity and robustness, sets these systems apart from true biocatalysts. By contrast, miniaturized enzymes retain key features of the protein scaffold, surrounding the metal center through short peptide sequences that reproduce both the primary coordination shell and the key secondary interactions, required to support catalysis while protecting the active site from degradation.^5^ These systems therefore lay at the interface between small-molecule and biological catalysts, crossing the boundaries between these two worlds for combining advantageous features of both. They are designed to preserve the high selectivity of metalloenzymes while offering the broader reactivity and synthetic versatility typically associated with small-molecule catalysts. Our group and others have applied this approach to develop artificial metalloproteins that include, but are not limited to, heme proteins. While the earliest heme protein models were mainly designed to explore specific aspects of heme protein properties,^72^ more recent efforts have converged on a single family of molecules with remarkable catalytic potential: mimochromes (MCs). MCs are composed of two short helical peptides, covalently linked to deuteroporphyrin IX (DPIX, Fig. 2). The two helices are designed to mimic the proximal and distal sites of natural heme proteins, from globins to peroxidases. By wrapping around the porphyrin in a helix–heme–helix sandwiched structure, they create an active site that is protected from bulk solvent while retaining sufficient accessibility for substrate binding and turnover. The structural simplicity of MCs has led to several milestones in the field of designed heme enzymes. First, by reducing the steric constraints imposed by a complex protein environment, MCs overcome the intrinsically narrow substrate specificity of natural systems, enabling substrate promiscuity.^73–77^ Second, they are readily accessible through chemical synthesis^78^ and can be easily modified to tailor their properties.^73,79–81^ Third, MCs operate efficiently under mild conditions, such as in aqueous solution at neutral pH and ambient temperature,^73,81,82^ while working even better in organic solvents,^74,83^ which are often required for the transformation of poorly water-soluble substrates.

Since the original design (MC1) was developed by our group in the late 1990s, targeted mutations have been introduced into the peptide sequences to incorporate additional functional elements and enhance catalytic performances.^73,79,84^ Furthermore, replacement of iron with other first-row transition metals has expanded the accessible reactivities beyond oxidation chemistry,^81,82,85^ while opening the way to new applications, including the use as a photosensitizer in a fully artificial, light-triggered electron transfer chain.^6^

This perspective focuses on the catalytic activity of MCs, from the initial introduction and systematic optimization of peroxidase activity to the development of the current benchmark catalyst. Attention will be given to the role of the MC scaffold in modulating the reactivity of different metal ions, including iron, manganese and cobalt. The most significant findings are discussed in comparison with natural enzymes and selected small-molecule systems reported in the literature, highlighting the effectiveness of the miniaturization strategy in generating versatile yet selective metalloenzymes.

## Toward MC6*a: from structure to activity

The original design of MC was inspired by the heme environment of human hemoglobin (PDB ID: 2HHB), from which a short nonapeptide fragment (Leu88–Leu96) was extracted to provide the proximal histidine ligand and to shield one face of the porphyrin.^5^ A covalent linkage between Lys95 and a propionic group of the porphyrin ensured stable peptide-cofactor attachment, while some residue substitutions were introduced to stabilize the structure in the absence of the outer protein matrix. The coupling of a second identical peptide chain to the other propionic group of DPIX gave rise to the mimochrome prototype, named MC1, with a characteristic helix–heme–helix sandwiched structure and a six-coordinate heme site.^5^

Starting from there, MCs have been optimized through iterative rounds of rational design, aimed at stabilizing the overall structure and engineering the catalytic activity.^86,87^ In this context, MC4 (Fig. 2) represented the first six-coordinate mimochrome analogue for which high-resolution structures were obtained by NMR^86^ and X-ray crystallography,^87^ providing key structural insights that guided the further evolution of the scaffold.

A five-coordinate metal center was introduced into MC6, mimicking the active site of peroxidases.^88^ Two peptide chains differing in length and composition were incorporated in MC6, a proximal tetradecapeptide (TD) providing an axial histidine ligand and a distal decapeptide (D) creating a substrate binding pocket, where Ser6(D) faces the metalloporphyrin, assisting hydrogen peroxide activation (Fig. 2).

**Fig. 3:**
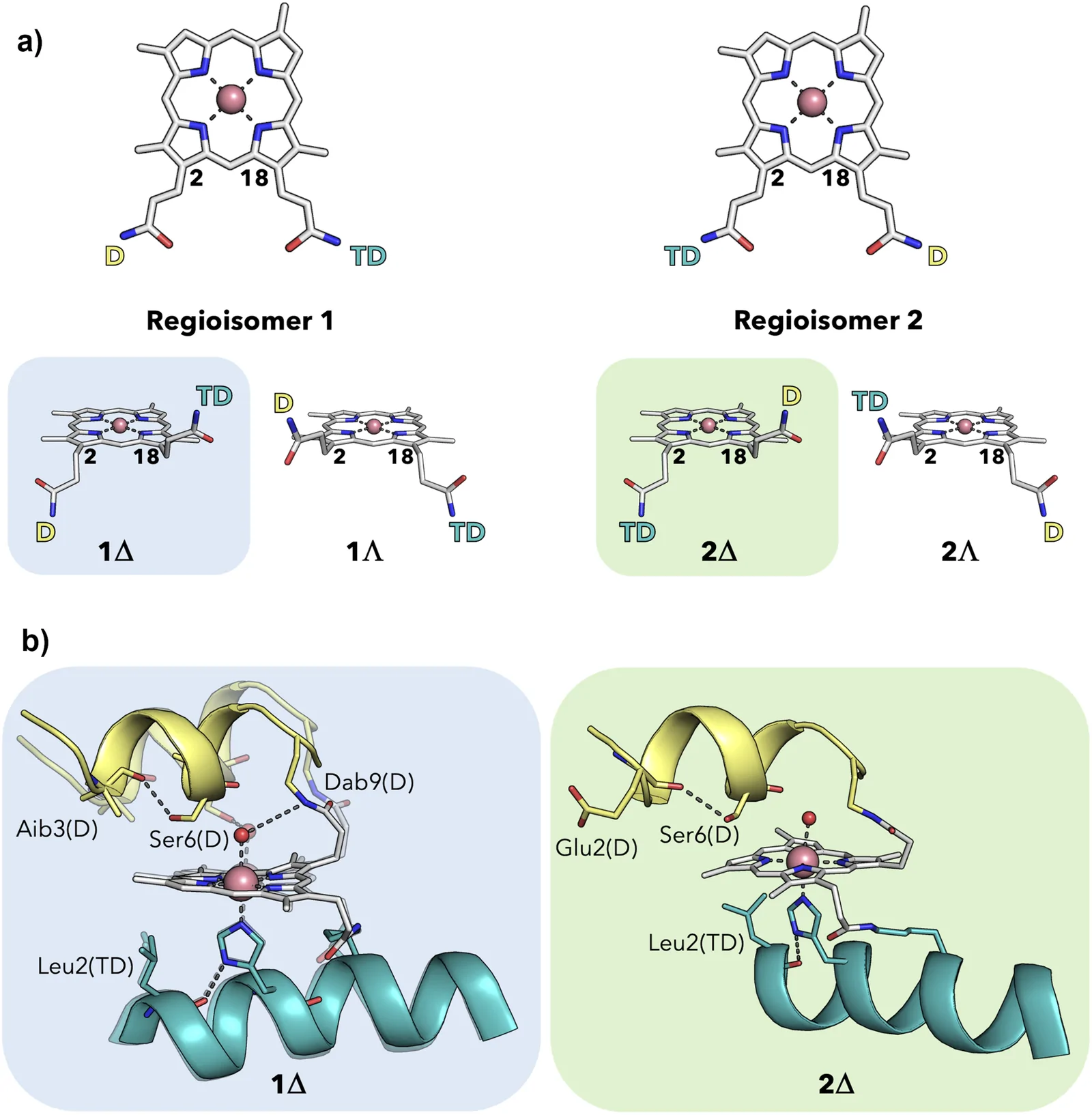
(a) Binding modes of D and TD peptide chains to the deuteroporphyrin propionate groups at positions 2 and 18, giving rise to regioisomers 1 and 2 in MC6, MC6* and MC6*a. For each regioisomer, Δ and Λ diastereoisomers are shown. (b) NMR structures of Co^III^Lys9DabMC6*a-1Δ (labelled as 1Δ, PDB ID: 9GSA) and Co^III^Lys9DabMC6*a-2Δ (labelled as 2Δ, PDB ID: 9GW6), showing the axial coordination and the second-shell interaction network. In both regioisomers, Co^III^ axial coordination occurs through the imidazole Nε atom, as already found for others Co^III^MCs complexes.^78,86^ In 1Δ, Ser6(D) hydrogen bonds to the Aib3(D) backbone carbonyl (75% occurrence) or to the Co^III^-bound hydroxide ligand (25% occurrence). Adapted from ref. 84 with permission from American Chemical Society, copyright 2026.

The asymmetric architecture of MC6 introduces an additional layer of structural complexity, as the attachment of the two different peptide chains to the porphyrin propionates may provide two regioisomeric species. Each of them adopts both Δ and Λ diastereomeric configurations, depending on the relative arrangement of the peptides around the porphyrin (Fig. 3a), as previously observed in the structurally characterized MC1 prototype.^78^ MC6 was the first analogue to display peroxidase activity in its Fe-complex (FeMC6), promoting the oxidation of the model chromogenic substrate ABTS (2,2′-azino-bis(3-ethylbenzothiazoline-6-sulfonic acid)) upon activation of H_2_O_2_ (Table 1, entry 1).^88^ Point mutations were then introduced into MC6 in two subsequent generations to enhance catalytic performance and robustness, giving rise to MC6* ^79^ and then to MC6*a (Fig. 2).^73^ The first modification involved the substitution of Glu2 with Leu2 in the proximal (TD) chain, thereby disrupting a Glu2(TD)-Arg10(D) salt bridge and allowing a higher conformational freedom to Arg10(D) (Fig. 2a). This increased mobility mirrors the behavior of the distal arginine in natural peroxidases,^89^ which polarizes the O–O bond and facilitate formation of the high-valent oxoferryl radical cation intermediate [(Fe^IV^=O)por˙^+^], analogous to Compound I (Cpd I) of natural peroxidases.^79^ As a result, FeMC6* exhibits a two-fold higher *k*_cat_ for ABTS oxidation and a nearly three-fold improvement in catalytic efficiency (*k*_cat_/*K*_m_) for both ABTS and H_2_O_2_ relative to FeMC6 (Table 1, entry 2). A second round of redesign strengthened the porphyrin–peptide interactions. Two polar residues (Gln3 and Ser7) in the distal (D) chain were replaced with the non-canonical amino acid α-aminoisobutyric acid (Aib), the simplest C^α,α^-disubstituted amino acid, to favor the helical folding in the D chain, enabling a tighter packing of the helix against the porphyrin.^73^ As a result, FeMC6*a displayed enhanced resistance to oxidative degradation, with its turnover number (TON = 14 000, Table 1, entry 3) more than doubling that of FeMC6* (TON = 5900, Table 1, entry 2). FeMC6*a retained catalytic efficiencies (*k*_cat_/*K*_m_ values for both H_2_O_2_ and ABTS) similar to those of its predecessor, arising from a higher *k*_cat_ and increased *K*_m_ values, consistent with a narrower and more hydrophobic distal site.^73^ A detailed structure–function correlation of MC6*a has long been hindered by its intrinsic structural heterogeneity, arising from multiple regio- and diastereomeric species in solution. This limitation has been recently overcome through a targeted mutation, in which the linker connecting the distal peptide to the porphyrin was shortened (Lys9DabMC6*a), enabling isolation and spectroscopic characterization of individual regioisomers (namely Lys9DabMC6*a-1 and Lys9DabMC6*a-2).^84,90^ Importantly, the resolved NMR structures of the diamagnetic Co^III^Lys9DabMC6*a-1 and Co^III^Lys9DabMC6*a-2, revealed that the two regioisomers (both in the Δ configuration, and thus termed as 1Δ and 2Δ in the following) exhibit different second-shell interactions involving the residues within the distal pocket (Fig. 3b), modulating both metal electronic properties and substrate accessibility.^84^ In particular, structural analyses have revealed an increased accessibility of the distal site for 1Δ, due to a more “open” orientation of the D chain compared to that observed in 2Δ. Further, while the Ser6(D) sidechain is only hydrogen-bonded with the Glu2(D) carbonyl backbone in 2Δ, it forms hydrogen bonds with either Aib3(D) carbonyl backbone or the Co^III^-bound hydroxide ligand in 1Δ, with an occurrence of 75% and 25%, respectively (Fig. 3b). These effects manifest in the divergent kinetic profiles of FeLys9DabMC6*a regioisomers in the ABTS oxidation. Indeed, FeLys9DabMC6*a-1 (Table 1, entry 4) displays improved substrate affinity (lower *K*_m_), consistent with a more accessible distal site, while FeLys9DabMC6*a-2 (Table 1, entry 5) shows enhanced catalytic turnover (higher *k*_cat_), suggesting a role of the serine residue in assisting H_2_O_2_ activation. These findings highlight how minimal structural perturbations can finely tune enzymatic performance.^90^

**Table 1:** Steady-state kinetic parameters for the H_2_O_2_-dependent oxidation of ABTS catalyzed by FeMC6 analogues, HRP, and selected *de novo* designed heme enzymes

Entry	Enzyme	*k*_cat_ (10^3^ s^−1^)	*K*_m_^ABTS^ (10^−2^ mM)	*K*_m_^H_2_O_2_^ (mM)	*k*_cat_/*K*_m_^ABTS^ (10^3^ mM^−1^ s^−1^)	*k*_cat_/*K*_m_^H_2_O_2_^ (10^3^ mM^−1^ s^−1^)	TON	Ref.
1	FeMC6	0.37 ± 0.01	8.4 ± 0.2	44 ± 2	4.4 ± 0.2	8.4 ± 0.4	4000	88
2	FeMC6*	2.3 ± 0.2	5.0 ± 0.6	130 ± 20	46 ± 7	0.018 ± 0.003	5900	79
3	FeMC6*a	5.8 ± 0.3	9 ± 1	440 ± 50	64 ± 8	0.013 ± 0.002	14 000	73
4	FeLys9DabMC6*a-1	1.00 ± 0.02	2.8 ± 0.1	204 ± 8	36 ± 2	0.0049 ± 0.0003	n.r.^a^	90
5	FeLys9DabMC6*a-2	3.0 ± 0.2	27 ± 2	234 ± 19	11 ± 2	0.013 ± 0.002	n.r.^a^	90
6	HRP	2.70 ± 0.03	70 ± 8	0.93 ± 0.05	3.8 ± 0.5	2.9 ± 0.2	50 000	73
7	CTM C45	1.2	37.9	94	3.2	0.013	n.r.^a^	29
8	FeMP3	0.535	34	172.5	1.573	0.0031	1000	92
9	Biot_2_-FePP·Sav	5.7	n.r.^a^	0.404	n.r.	14.1	n.r.^a^	60

a n.r.: not reported.

Remarkably, FeMC6*, FeMC6*a, and FeLys9DabMC6*a exhibit *k*_cat_/*K*_m_ values toward ABTS surpassing that of the natural horseradish peroxidase (HRP, Table 1, entry 6), while their catalytic efficiencies toward H_2_O_2_ are markedly lower. This discrepancy further confirms that HRP has evolved to recognize and activate hydroperoxides, while binding organic substrates less tightly.^91^ In contrast, MCs display a higher affinity for the reducing co-substrates, such as ABTS, consistent with a more accessible active site, which is not readily saturated by small molecules like H_2_O_2_, but able to accommodate bulkier substrates. Accordingly, HRP remains a more efficient catalyst for H_2_O_2_ reduction, whereas its MC mimics, in particular FeMC6*a, are among the most efficient catalysts for ABTS oxidation (*k*_cat_/*K*_m_^ABTS^), when compared to HRP (Table 1, entry 6) and other designed peroxidase catalysts (Table 1, entries 7–9). In this respect, artificial systems featuring more elaborate protein scaffolds generally display a higher affinity for H_2_O_2_ than FeMC6*a. This is exemplified by the *de novo* four-helix bundle C45 (Table 1, entry 7)^29^ and, even more strikingly, by the artificial metalloenzyme Biot_2_-FePP·Sav (Table 1, entry 9).^60^ Indeed, the larger protein framework enables the presence of a more extensive network of interactions around the cofactor, favoring peroxide recognition and activation. In the latter case, structure-guided genetic optimization of the streptavidin scaffold afforded a catalyst displaying a *K*_m_^H_2_O_2_^ value (0.404 mM) even lower than that of HRP, together with the highest *k*_cat_/*K*_m_^H_2_O_2_^ value among the systems considered here. Nevertheless, FeMC6*a^73^ and Biot_2_-FePP·Sav^60^ display similar *k*_cat_ values (5.8 × 10^3^ s^−1^*vs.* 5.7 × 10^3^ s^−1^), demonstrating that highly efficient turnover can be achieved even within a much simpler miniaturized scaffold.

The activity of MCs relies on the presence of 2,2,2-trifluoroethanol (TFE) as a co-solvent,^93^ which enables the transformation of substrates with limited aqueous solubility. As widely known, the effect of TFE on metalloprotein structure is strongly dependent on the nature and structural organization of the protein scaffold. In natural globular metalloproteins, TFE can initially stabilize secondary-structure elements and modulate protein dynamics, whereas at higher concentrations it may disrupt tertiary packing and promote partial unfolding.^93^ As an example, in HRP, low TFE concentrations (<25%) enhance activity, whereas higher amounts (>40%) disrupt tertiary structure leading to loss of activity.^94^ In *de novo* and miniaturized metalloenzymes, which often lack the extensive tertiary interactions of natural proteins, TFE can favor the formation and stabilization of α-helical structures, while concurrently affecting the equilibrium of conformational states, pointing to a more globally rigid and preorganized metal-binding environment.^95^ As an illustrative example, temperature-dependent studies on the artificial peroxidase CTM C45 demonstrated a beneficial role of TFE (up to 80%) on both catalytic rate (*k*_cat_) and robustness (TON), which was attributed to an increased scaffold rigidity.^96^ A similar role of TFE can be reasonably proposed for MCs. Owing to their smaller size compared to C45, TFE exerts a more pronounced effect on promoting helical folding in MCs. This effect likely leads to a marked preorganization of the scaffold, which strengthens porphyrin–peptide interactions and stabilizes the active-site architecture. Notably, this effect parallels the strategy adopted in the design of MC6*a, where the introduction of Aib residues was intended to favor helical folding of the distal chain and its interactions with the cofactor. Interestingly, the use of TFE also plays a crucial role in simplifying the conformational heterogeneity of MC6 systems. CD studies on the isolated Δ and Λ diastereomers of MC1 displayed negative and positive Cotton effects in the Soret band region, respectively, reflecting opposite distortions of the porphyrin.^78^ The negative Cotton effect observed at high TFE concentrations (50%) for FeMC6*a^73^ is therefore consistent with the preferential stabilization of the Δ isomer. This effect suggests that TFE may amplify design-encoded features of the MC6*a scaffold.

## Benchmark peroxidase activity: FeMC6*a-catalyzed oxidation of halophenols

FeMC6*a stands among the most efficient peroxidase catalysts reported to date for the oxidation of a broad range of substrates beyond ABTS, including luminol,^75^ organic dyes^76^ and halogenated phenols.^74,77,97^ Among these transformations, the oxidation of halophenols has been investigated in greater detail. Beyond its relevance to environmental remediation, this reaction provides a valuable model for elucidating how substrate substitution, including both the nature and number of halogen substituents, affects activity and selectivity. The oxidation of 2,4,6-trichlorophenol (TCP) is the most extensively investigated reaction, which was comparatively evaluated using both natural and artificial heme enzymes. In this context, FeMC6*a promotes the oxidative dehalogenation of TCP to 2,6-dichlorobenzoquinone (DCBQ) demonstrating remarkable robustness, as highlighted by the TON value of ∼3500.^74,97^ In addition, steady-state kinetic analysis revealed that FeMC6*a is among the most catalytically efficient systems reported to date for this reaction^74^ (Table 2). The enzyme exhibits a turnover frequency (Table 2, entry 1) that is 4-fold higher than HRP, the most active natural metalloenzyme^98^ (Table 2, entry 3), and nearly 90-fold higher than the best engineered myoglobin variant^99^ (F43Y/H64D Mb, Table 2, entry 6). Moreover, the Michaelis–Menten constant for TCP (Table 2, entry 1) is almost 350-fold lower than that of HRP (Table 1, entry 3), indicating a considerably higher substrate affinity. This finding is consistent with the results obtained in the ABTS oxidation and further supports the idea that the miniaturized enzyme can accommodate a broad range of substrates, whereas HRP is evolutionarily optimized to bind hydrogen peroxide with extremely high affinity.^91^ Notably, FeMC6*a (Table 2, entry 1) displays a *K*_m_ for TCP that is even lower than that reported for DHP B (Table 2, entry 5), a natural enzyme evolved for catalyzing this reaction. This seemingly counterintuitive observation can be explained by the dual functional role of DHP B, which acts both as a respiratory globin and a peroxidase.^100^ Such functional promiscuity likely imposes structural and dynamic constraints on the active site: it must retain sufficient flexibility for oxygen binding, which in turn limits the optimization of a highly specific pocket for TCP binding. In contrast, the miniaturized scaffold of FeMC6*a, lacking these evolutionary trade-offs, provides a more accessible and adaptable binding environment that enhances substrate affinity. Because of its low *K*_m_ and remarkably high *k*_cat_, FeMC6*a displays a catalytic efficiency (Table 2, entry 1) that exceeds that of HRP (Table 2, entry 3) by 1400-fold and that of F43Y/H64D Mb (Table 2, entry 6) by about 20-fold. Even in the absence of TFE, FeMC6*a retains remarkable activity (Table 2, entry 2), outperforming DHP A (Table 2, entry 4), DHP B (Table 2, entry 5), HRP (Table 2, entry 3), and the *de novo* designed peroxidase CTM C45 (Table 2, entry 8), while being surpassed only by F43Y/H64D Mb (Table 2, entry 6).

**Table 2:** Steady-state kinetic parameters for the H_2_O_2_-dependent TCP dehalogenation by natural and artificial heme enzymes

Entry	Enzyme	*K*_m_^TCP^ (µM)	*K*_m_^H_2_O_2_^ (mM)	*k*_cat_ (s^−1^)	*k*_cat_/*K*_m_^TCP^ (mM^−1^ s^−1^)	Ref.
1	FeMC6*a^a^	16 ± 2	120 ± 10	2400 ± 80	150 000	74
2	FeMC6*a^b^	100 ± 21	94 ± 8	70 ± 6	700	74
3	HRP	5400	0.05	571	105.8	98
4	DHP A	2070	0.34	13.7	6.6	98
5	DHP B	685	0.17	25.7	37.5	98
6	F43Y/H64D Mb	3.5 ± 0.3	n.r.^c^	27.4 ± 0.9	7828	99
7	A15C/H64D Ngb	130 ± 10	n.r.^c^	43.9 ± 0.6	340	101
8	CTM C45	12	n.r.^c^	0.50	41.7	29

a Experimental conditions: [enzyme] = 6.7 × 10^−8^ M; 50 mM phosphate buffer, pH 6.5 with 50% (v/v) TFE.

b [Enzyme] = 6.7 × 10^−8^ M; 50 mM phosphate buffer, pH 6.5.

c n.r. = not reported.

In addition to TCP, FeMC6*a is able to oxidize a broad range of halophenols, including mono-, di-, tri- and penta-substituted derivatives,^74,77,98^ although attention has been mainly focused on the 4-halophenol series (4-XPs, X = F, Cl, Br, I), which act as inhibitors of both DHP A and DHP B.^102,103^ Interestingly, distinct product distributions are observed depending on the halogen substituent. While 4-fluorophenol (4-FP) exclusively undergoes oxidative dehalogenation to yield 1,4-benzoquinone (BQ), 4-chlorophenol (4-CP), 4-bromophenol (4-BP), and 4-iodophenol (4-IP) predominantly afford C–O bond-forming oligomerization products, with a chemoselectivity between the oxidative dehalogenation and the oligomerization reaction up to 95%.^74,77^

**Scheme 1:**
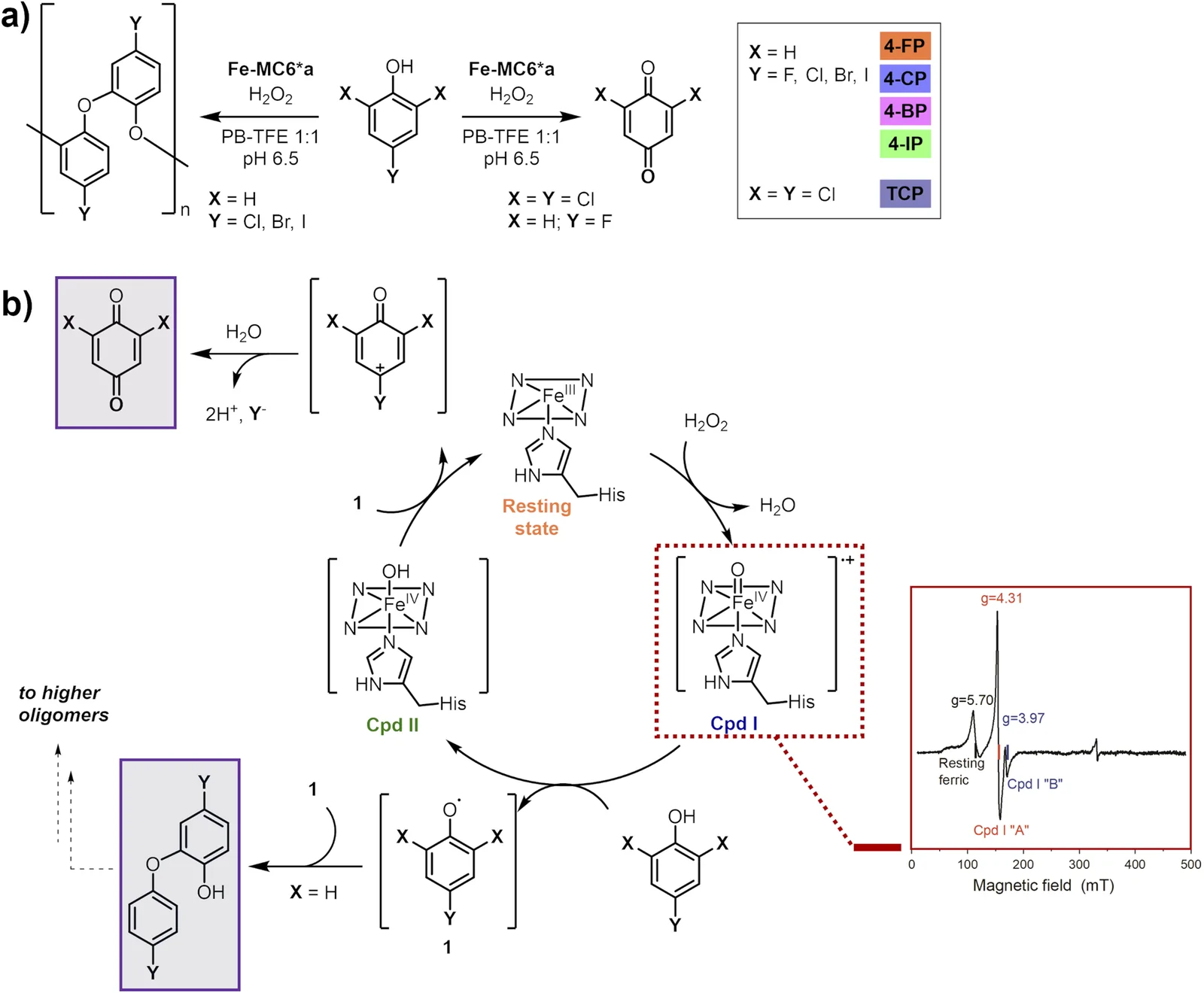
(a) Chemoselective halophenol oxidation catalyzed by FeMC6*a. (b) Reaction mechanism explaining the origin of chemoselectivity based on the accumulation of radical species 1. Reproduced from ref. 97 with permission from Elsevier, copyright 2026. The inset highlighted in red shows the EPR spectra of FeMC6*a Cpd I. Adapted from ref. 74 with permission from Royal Society of Chemistry, copyright 2022.

The kinetic parameters of FeMC6*a in 4-FP oxidation are comparable to those observed for TCP oxidation,^97^ with nearly identical *k*_cat_ values (2400 for TCP *vs.* 2100 s^−1^ for 4-FP), and a 3-fold higher *K*_m_ for 4-FP (*K*_m_^4-FP^ = 52 ± 5 µM). This results in a catalytic efficiency for 4-FP oxidation (*k*_cat_/*K*_m_ ∼ 40 400 mM^−1^ s^−1^) that is approximately 3-fold lower than that measured for TCP, although still higher than those reported to date for all natural peroxidases.^97^ The peculiar behavior of FeMC6*a in halophenol oxidation has prompted detailed mechanistic investigations. Spectroscopic analyses suggest that the FeMC6*a-catalyzed oxidation of halophenols proceeds *via* a two-step, single electron oxidation pathway as depicted in Scheme 1. Upon reaction with H_2_O_2_, the resting ferric species of FeMC6*a forms Cpd I [(Fe^IV^=O)por˙^+^], which promotes the one-electron oxidation of the halophenol to the corresponding phenoxyl radical. This intermediate can then undergo a second oxidation by Cpd II (a Fe^IV^ form lacking the porphyrin radical), yielding a positively charged dienone, which is subsequently converted to the corresponding benzoquinone *via* water-assisted halide displacement. This mechanism predominates in the case of TCP and 4-FP, leading to the formation of DCBQ and BQ, respectively. Alternatively, self-coupling of phenoxyl radicals competes effectively for 4-halophenols other than 4-FP, providing dimeric and oligomeric products. EPR studies on TCP oxidation have provided direct insight into the catalytic cycle,^74^ allowing characterization of the reactive intermediates. In the resting state, FeMC6*a exhibits signals typical of a pentacoordinate high-spin Fe^III^ center. Upon reaction with H_2_O_2_, two spectroscopically distinct species (Cpd I “A” and Cpd I “B”) appear (Scheme 1).^74^ Although in principle they could correspond to either regio- or diastereoisomers of the activated catalyst, their association to the Δ and Λ forms of FeMC6*a appears a more plausible hypothesis. Indeed, the integration of the EPR signals of Cpd I “A” and Cpd I “B” indicates a prevalence for Cpd I “A”, matching with the preferred occurrence for the Δ isomer as resulting from CD data.^5,73^

When sub-stoichiometric TCP (0.5 eq.) was added to Cpd I, approximately half the sample becomes EPR-silent, consistent with formation of Cpd II. With stoichiometric TCP (1 eq.), complete conversion to resting state occurs without detectable Cpd II accumulation, consistent with fast kinetics and rate-limiting decay of Cpd I.

Spin-trapping and EPR experiments following 4-halophenols oxidation also provided direct evidence for the chemodivergent behavior of FeMC6*a.^97^ Accordingly, 4-CP oxidation leads to the formation and accumulation of 4-chlorophenoxyl radical, which undergo bimolecular coupling to form a dimeric species, which subsequently evolves into oligomeric products. In contrast, no significant radical accumulation was observed during 4-FP oxidation, as they are rapidly converted to BQ. The origin of this chemodivergent outcome is not yet established and is currently under investigation. As fluorine is a stronger π-electron donor than chlorine by resonance, the 4-fluorophenoxyl radical intermediate might be expected to be more effectively stabilized than its chlorinated counterpart. This difference in radical stabilization could in principle account for the distinct reactivity observed for the two substrates. However, a reference 4-halophenol oxidation reaction promoted by hexacyanoferrate(III) afforded comparable 4-fluoro- and 4-chlorophenoxyl radicals.^97^ This result indicated that the electronic properties of the substrates do not dictate product distribution, likewise suggesting that the enzyme itself plays an active role in determining selectivity. We favor the hypothesis that FeMC6*a processes 4-FP and 4-CP differently because Cpd II converts the 4-fluorophenoxyl radical to the corresponding cationic dienone intermediate more rapidly than 4-chlorophenoxyl radical. The slower conversion of the latter would be expected to promote its accumulation, thereby favoring the observed self-coupling reaction.

Beyond its substrate-dependent chemoselectivity, FeMC6*a also exhibits an unusual functional duality, as 4-halophenols were found to simultaneously act as substrates and as competitive inhibitors of TCP dehalogenation.^97^ This behavior markedly differs from that of the natural dehaloperoxidases DHP A and DHP B, where 4-halophenols act exclusively as inhibitors.^102,103^ In mixed-substrate competition assays, all 4-halophenols significantly reduced the FeMC6*a-catalyzed TCP conversion, although the enzyme retained measurable catalytic activity under all conditions. This partial tolerance to inhibition suggests a potential applicability of FeMC6*a in wastewater treatment, where several halophenol contaminants may coexist. Reciprocal inhibition experiments highlighted that TCP also reduced 4-FP turnover, although to a lesser extent. This behavior reflects different substrate affinities, consistent with their different *K*_m_ values, and indicates that the enzyme preferentially oxidizes TCP also in the presence of competing substrates, such as 4-FP.

## Tuning reactivity through metal exchange: peroxygenase activity of MnMC6*a

Metal cofactor replacement in metalloproteins generally requires removal of the endogenous cofactor followed by reconstitution with an alternative one.^15^ In contrast, this is considerably easier in synthetic systems such as MCs, where metal insertion represents the final step of the synthetic procedure. In principle, any metal ion capable of being coordinated by the DPIX ligand can be introduced. For MC6*a, the first explored metal substitution consisted in swapping iron with manganese.^80^ Due to their proximity in the periodic table, Fe^III^ and Mn^III^ share similar size and coordination preferences, although Mn^III^ is characterized by slightly lower Lewis acidity and more positive Mn^III^/Mn^II^ reduction potentials compared to Fe^III^/Fe^II^. Mn-substitution in heme proteins and metalloporphyrins usually results in stabilized high-valent intermediates compared to the iron counterparts,^104,105^ likely resulting in decreased reactivity.^106^ At the same time, Mn-porphyrins can more easily access the Mn^V^ oxidation state.^106,107^ The improved stability of the Mn-oxo species compared to their iron counterparts can be conceivably attributed to the lower electronegativity and Lewis acidity of Mn compared to Fe, which results in a reduced polarization of the Mn–O bond in Mn-oxo species. In contrast, the more electronegative and acidic Fe center strongly polarizes the Fe–O bond, increasing the electrophilic character of the oxo moiety and, consequently, the reactivity of the resulting Fe-oxo species.

MnMC6*a displayed spectroscopic properties resembling those of Mn-substituted HRP (Mn-HRP).^108^ Similarly to Mn-HRP, MnMC6*a was able to activate H_2_O_2_ forming a high-valent species, identified as a Mn-analogue of Cpd I (formally a Mn^IV^-oxo species with a porphyrin radical, [(Mn^IV^=O)por˙^+^]).^80^ As a striking difference, while this species was remarkably long-lived (persisting for days) in Mn-HRP, it rapidly decayed (within minutes) back to the Mn^III^ resting state in MnMC6*a, when in the absence of external substrates. The catalytic activity of MnMC6*a was first evaluated in the oxidation of thioanisole and other phenyl thioethers, where it demonstrated the ability to promote sulfoxidation of its substrates by Mn-Cpd I. Under optimized conditions, MnMC6*a achieved up to 800 TON in thioanisole oxidation, which remains among the highest values ever reported for metalloporphyrin-based natural or artificial enzymes.^80^ The only catalyst that outperforms MnMC6*a in terms of TON is FeMC6*a, which reaches up to 1500 turnovers. As expected, the time required for achieving complete substrate conversion was strongly influenced by pH. The pH-dependent thioanisole oxidation by MnMC6*a correlated with the rate and yield of Mn-Cpd I formation. These parameters reached their maximum values at pH 10, whereas FeMC6*a exhibits optimal catalytic performance at pH 6.5. These differences reflect the lower ability of manganese compared to iron in lowering the p*K*_a_ of H_2_O_2_. Thus, a higher OH^−^ concentration is needed for assisting H_2_O_2_ deprotonation and cleavage. The same reaction mechanism was proposed for both Fe and MnMC6*a, involving direct oxygen atom insertion into the substrates, as supported by isotopic labelling experiments.^80^ Notably, these findings indicate that substitution of manganese with iron did not dramatically alter the intrinsic reactivity of MC6*a. The lower TON achieved by MnMC6*a compared to FeMC6*a at their optimal conditions suggests that alternative pathways may contribute to H_2_O_2_ consumption, resulting in a larger number of unproductive catalytic cycles. It is reasonable to hypothesize that MnMC6*a may facilitate H_2_O_2_ disproportionation at alkaline pH values, a process that could compete with productive substrate sulfoxidation. However, the ability of the two metal derivatives to operate optimally at complementary pH values effectively broadens the pH range over which these catalysts can be employed.^80^

## Selective indole oxidation by MnMC6*a

As the ability of MnMC6*a to promote oxygenation reactions was assessed, its catalytic properties were tested in a reaction relevant to synthetic chemistry: indole oxidation.^83^ This transformation represents a significant challenge for selectivity, as most natural heme enzymes have failed to provide single reaction products. Most research in this context has focused on the biocatalytic synthesis of indigo from indole. However, increasing attention has also been devoted to 2-oxindole and 3-oxindole derivatives, because of their biological potential and their utility as versatile building blocks for the synthesis of pharmacologically active molecules. Indeed, the oxindole scaffold represents a recurring pharmacophore in medicinal chemistry, with applications spanning a wide range of therapeutic areas, including cancer, neurodegeneration, metabolic disorders and infectious diseases.^109–112^ The challenge to achieve selectivity lies in the inherent reactivity of indole. Unlike aryl-substituted olefins such as styrene, the C2–C3 double bond of indole is part of a heteroaromatic system. Besides undergoing a conventional epoxidation pathway, indole is prone to oxidation at the C3 position, which is the most nucleophilic site of the molecule due to the electron-donating effect of the nitrogen atom. While epoxidation typically leads to the formation of the stable 2-oxindole, oxidation at the C3 position results in the formation of 3-indoxyl (in equilibrium with 3-oxindole), a highly reactive species which further undergoes spontaneous oxidation and/or dimerization. The coexistence of these competing pathways accounts for the complex product distributions observed when natural heme enzymes are used as catalysts.^113–115^ Selective indole oxidation at C3 position is particularly relevant, as it underpins the biocatalytic synthesis of indigo, which is obtained by 3-oxindole dimerization.^116^ Among heme enzymes, only the unspecific peroxygenase from *Hypoxylon* sp. EC38 achieves the highly selective formation of indigo (98% yield, 2800 TON),^117^ while other thiolate-bound heme enzymes,^118–120^ such as cytochromes P450 and chloroperoxidase (CPO), predominantly form 2-oxindole, in line with an epoxidation mechanism. This mechanistic picture is reinforced by investigations of biomimetic high-valent iron-oxo porphyrins, which established epoxidation of the C2–C3 bond, followed by a 2,3-hydride migration, as the key pathway leading selectively to 2-oxindoles.^121^

Protein engineering has delivered notable improvements in selectivity towards indole C3 oxidation. The F43Y Mb mutant reaches 80% selectivity toward indigo formation,^122^ while a peroxygenase obtained *via* stepwise evolution of the P450BM3 heme domain enables the efficient synthesis of indigoid dyes (up to 80% yield, 6588 TON).^123^ In contrast, metal ion substitution in heme enzymes has not been explored for indole oxidation. MnMC6*a therefore represents the first example from both natural and artificial manganese-based enzymes investigated so far for this transformation. Previous insight from small-molecule metalloporphyrins^124,125^ had indicated that Mn^III^-porphyrins preferentially provide 2-oxindole (64%), whereas Fe^III^-porphyrins produce indigo in moderate amounts (20%), following distinct mechanistic pathways.^125^ In our hands, MnMC6*a exhibited unprecedented selectivity, producing a 3-oxindole derivative, namely 2-TFE-3-oxindole, as the main reaction product (up to 91% selectivity).^83^ This finding is notable for two reasons. First, it reveals an additional role for TFE in reactions catalyzed by MCs: beyond promoting peptide folding, TFE actively participates in the reaction mechanism.

Indeed, the presence of TFE enables trapping of a highly reactive species through derivatization of 3-oxindole at the C2 position, preventing the formation of indigo. This mechanism is reminiscent of that proposed for a flavin dependent Baeyer–Villiger monooxygenase, in which cysteine forms a covalent adduct with 3-oxindole, ultimately promoting indirubin formation *via* condensation with 2-oxindole.^126^

**Scheme 2:**
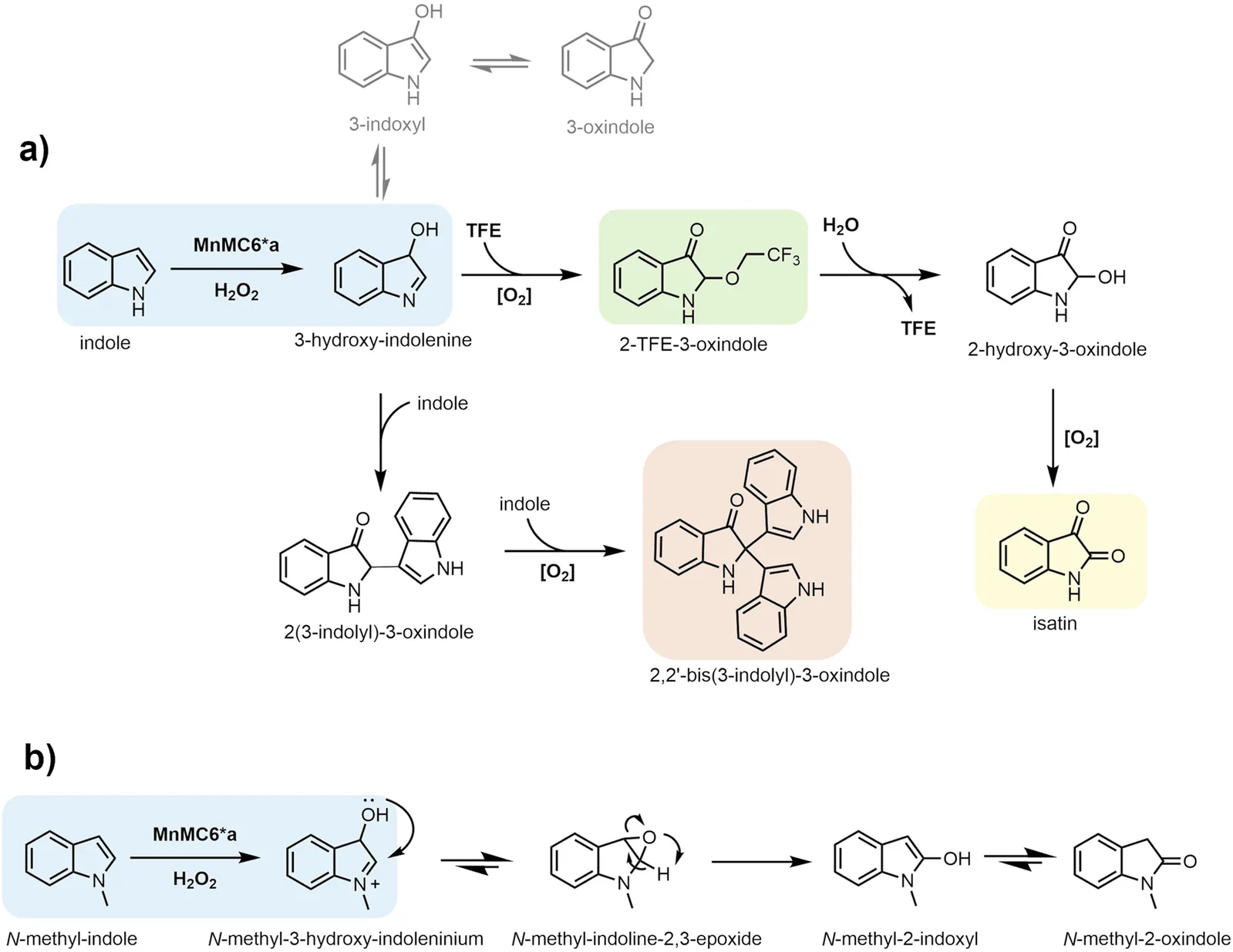
Proposed reaction pathway for the MnMC6*a-catalyzed oxidation of (a) indole and (b) *N*-methyl-indole with H_2_O_2_. As shown in panel (a), selective C3 oxidation of indole affords 3-hydroxy-indolenine (light blue), a key intermediate in equilibrium with the tautomeric forms 3-indoxyl and 3-oxindole (grey). Nucleophilic addition of TFE at C2, followed by aerobic oxidation, yields 2-TFE-3-oxindole (green), the main product observed at pH 8.5. Alternatively, reaction of 3-hydroxy-indolenine with unreacted indole and subsequent oxidation affords the pseudo-trimeric product 2,2′-bis(3-indolyl)-3-oxindole (orange), which is predominant at pH 6.5. Substitution of TFE by water in 2-TFE-3-oxindole generates 2-hydroxy-3-oxindole, which upon further oxidation gives isatin (yellow), the major product detected at pH 10. Panel (b) shows the selective C3 oxidation of *N*-methyl-indole (light blue), followed by intramolecular epoxidation and rearrangement to afford *N*-methyl-2-oxindole as the final product. Adapted from ref. 83 with permission from American Chemical Society, copyright 2021.

Second, it demonstrates a clear preference towards the C3-oxidation pathway (Scheme 2a). The proposed mechanism involves indole oxygenation mediated by Mn-Cpd I, as in the case of thioether sulfoxidation.^80^ The latter may occur through either a direct oxygen transfer or a stepwise “oxygen-rebound” mechanism. The origin of the observed selectivity is yet not fully understood. While the peptide scaffold is essential for enabling the catalytic activity of the Mn center, it does not appear to impose a specific substrate orientation that would account for the preferential oxidation at C3. Indeed, given the small and solvent-accessible nature of the active site, specific substrate–catalyst interactions are unlikely to be the primary determinant of selectivity. Instead, the peptide scaffold may modulate the electronic properties of the Mn center, thereby favoring a C3-hydroxylation over alternative oxidation pathways. A plausible explanation is that Mn-Cpd I of MnMC6*a is intrinsically less reactive than Cpd I of thiolate-ligated heme enzymes such as CPO, the latter exhibiting a clear preference for the epoxidation mechanism.^119,120^

Interestingly, product distribution was strongly influenced by pH. The highest selectivity toward 2-TFE-3-oxindole was observed at pH 7.5, although with moderate substrate conversion (Table 3, entry 1). Optimized conditions for isolating 2-TFE-3-oxindole were identified at pH 8.5, where a 57% substrate conversion was observed after 5 min, retaining high selectivity (Table 3, entry 2). At pH 10.5, substrate conversion further increased, consistent with the enhanced formation and reactivity of Mn-Cpd I under alkaline conditions (Table 3, entry 3). Indole-2,3-dione (isatin) was isolated as the main product, likely due to hydroxide-promoted substitution of the trifluoroethoxy group in 2-TFE-3-oxindole to form 2-hydroxy-3-oxindole, followed by spontaneous oxidation at C2 position. In contrast, at pH 6.5 the overall yield was lower in line with the reduced formation of Mn-Cpd I (Table 3, entry 4). Moreover, the lower nucleophilicity of TFE under these conditions allows unreacted indole to compete more effectively for reaction with 3-hydroxy-indolenine (Scheme 2), leading to the preferential formation of the pseudo-trimer 2,2-bis(3′-indolyl)-3-oxindole (Table 3, entry 4).

**Table 3:** Prevalent products observed in the oxidation of indole and *N*-methyl-indole promoted by MnMC6*a^83^^a^

Entry	Substrate	pH	Substrate conversion^b^ (%)	Prevalent product	Product selectivity^b^ (%)
1	Indole	7.5	27	2-TFE-3-oxindole	91
2	Indole	8.5	57	2-TFE-3-oxindole	86
3	Indole	10.5	100	Isatin	22
4	Indole	6.5	53^c^	2,2-Bis(3′-indolyl)-3-oxindole	n.d.^d^
5	*N*-Methyl-indole	8.5	54	*N*-Methyl-2-oxindole	n.d.^d^

a Experimental conditions: 10 µM MnMC6*a, 1 mM substrate, 1 mM H_2_O_2_; 50 mM phosphate buffer with 40% (v/v) TFE.

b Substrate conversion and product selectivity were measured after 5 minutes of reaction.

c Substrate conversion was measured after 60 minutes of reaction.

d n.d. = not determined.

Product selectivity of MnMC6*a is eventually altered upon substrate substitution. Indeed, oxidation of *N*-methyl indole in the optimized conditions found for indole oxidation (pH 8.5, 40% TFE) leads to *N*-methyl-2-oxindole as prevalent product (Table 3, entry 5).^83^ This finding remains consistent with the proposed mechanism of indole oxidation by the MC enzyme. Although this outcome may appear counterintuitive, *N*-methyl-2-oxindole arises from an initial oxidation at the C3 position, generating, in this case, the positively charged *N*-methyl-3-hydroxyindoleninium intermediate. This hypothesis is consistent with the increased nucleophilic character at C3 position of *N*-methyl-indole compared to indole, resulting from the electron-donating effect of the methyl group. Similarly, the increased electrophilicity at the C2 position, due to proximity of the positively charged nitrogen in *N*-methyl-3-hydroxyindoleninium, favors intramolecular nucleophilic attack by the 3-hydroxyl group over the addition of TFE, leading to the epoxide and ultimately to *N*-methyl-2-oxindole formation (Scheme 2b).

**Scheme 3:**
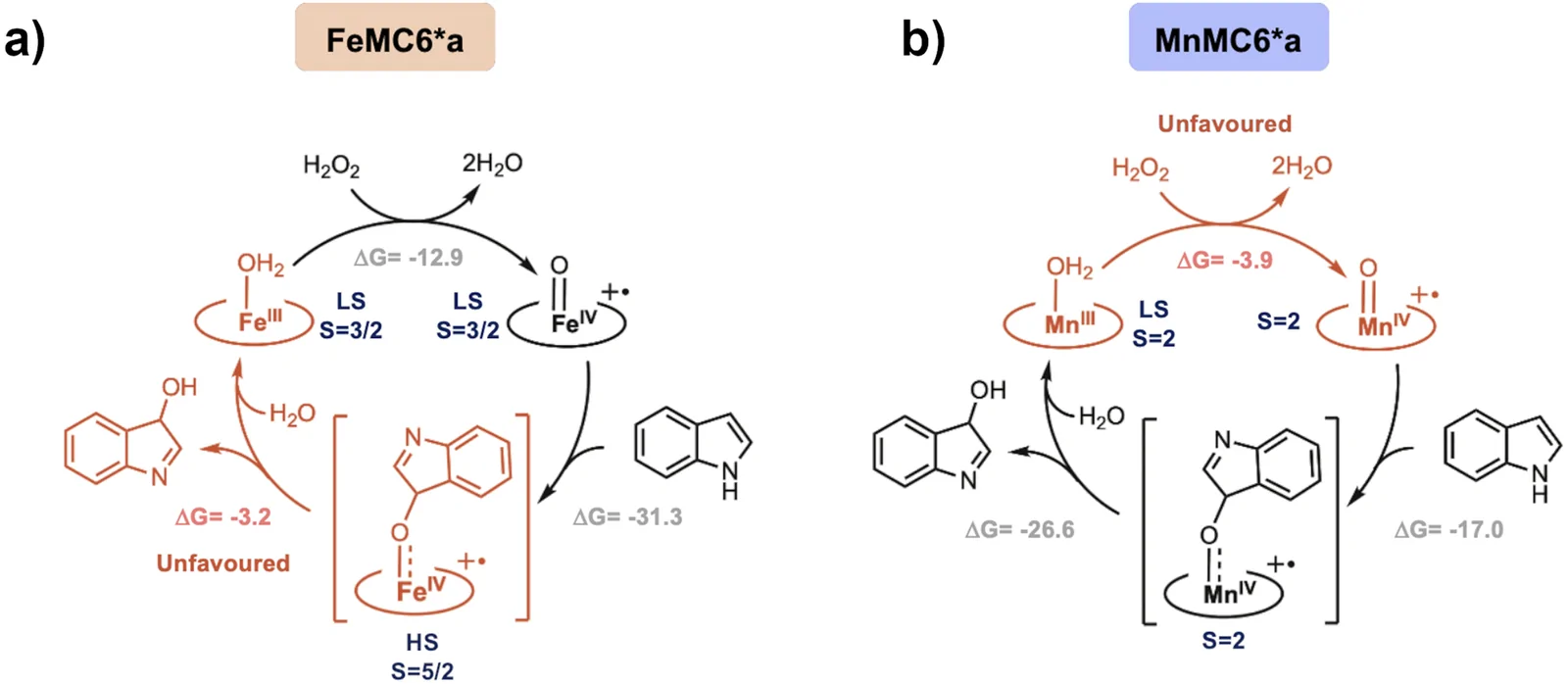
Proposed catalytic cycle for indole oxidation promoted by (a) FeMC6*a and (b) MnMC6*a, with the thermodynamic limiting step highlighted in red. For simplicity, the porphyrin is represented as a circle, and the axial histidine is not shown. Computed reaction free energies Δ*G* are expressed in kcal mol^−1^. Total spin (S) of species involved in the catalytic cycle and, if applicable, lowest-lying spin states (HS = high spin, LS = low Spin) are indicated. Adapted from ref. 127 with permission from Elsevier, copyright 2023.

A complementary set of experiments was performed to elucidate the role of the metal ion (Fe *vs.* Mn).^127^ The reaction catalyzed by FeMC6*a displayed the same chemoselectivity observed for MnMC6*a, leading to the formation 2-TFE-3-oxindole, although with a significantly lower yield. Conversely, oxidation of *N*-methyl-indole by FeMC6*a proceeded with a much higher conversion but without any selectivity, resulting in a complex mixture of products. Experimental and theoretical studies allowed us to reconstruct the reaction free energy profiles for both systems, revealing distinct thermodynamic limiting steps depending on the nature of the metal center (Scheme 3).^127^ DFT calculations highlighted that the Fe^IV^ center from FeMC6*a Cpd I switches from a d^4^ low-spin to a high-spin configuration upon reaction with indole, forming a [Fe–O–indole] intermediate which is unwilling to return to the resting ferric state, thus limiting substrate turnover (Scheme 3a). In the case of *N*-methyl-indole oxidation, the formation of a stable [Fe–O–indole] intermediate could favor inter-molecular nucleophilic attack at C2 position (by either H_2_O, TFE, or other substrate/product molecules) over intra-molecular epoxidation, ultimately leading to the observed complex product distribution. Conversely, in the case of MnMC6*a, release of the C3-hydroxylated product is more energetically favored than the formation of [Mn–O–indole] complex, which is likely not accumulating during turnover (Scheme 3b).^127^

Overall, these findings highlight the selectivity of the miniaturized mimochrome scaffold, which is achieved in the absence of a complex protein environment. In addition, structural simplicity of the MC scaffold makes it suitable for computational analyses which are difficult to perform when studying more complex metalloproteins, providing valuable insights into the mechanism of metalloporphyrin-catalyzed indole oxidation.

## Fueling catalysis: CoMC6*a for energy-relevant transformations

Cobalt complexes have received considerable attention as catalysts for the hydrogen evolution reaction (HER: 2H^+^ + 2e^−^ ⇌ H_2_).^128–132^ Although cobalt is not present in natural hydrogenase enzymes, which instead feature iron- and/or nickel-dependent cofactors (namely mononuclear Fe and dinuclear Fe–Fe or Ni–Fe centers), it is particularly well suited for this type of reactivity. Indeed, cobalt-containing complexes generally exhibit higher tolerance than iron to O_2_,^133^ owing to the intrinsic resistance of cobalt to oxidation, resulting from its high charge-to-radius ratio in the +3-oxidation state. Accordingly, substantial efforts have been devoted to the design of biomimetic cobalt complexes as functional alternatives to natural hydrogenases for scalable H_2_ production.

Starting from small-molecule coordination complexes, researchers have progressively increased structural complexity around the catalytic center,^130,134^ culminating in the incorporation of cobalt-containing cofactors into natural and artificial protein scaffolds. This introduces key functionalities for efficient H_2_ evolution, including hydrogen-bonding networks that facilitate proton shuttling to the active site, while the peptide/protein scaffold enhances water solubility and provides steric protection, thereby improving catalyst durability.

**Fig. 4:**
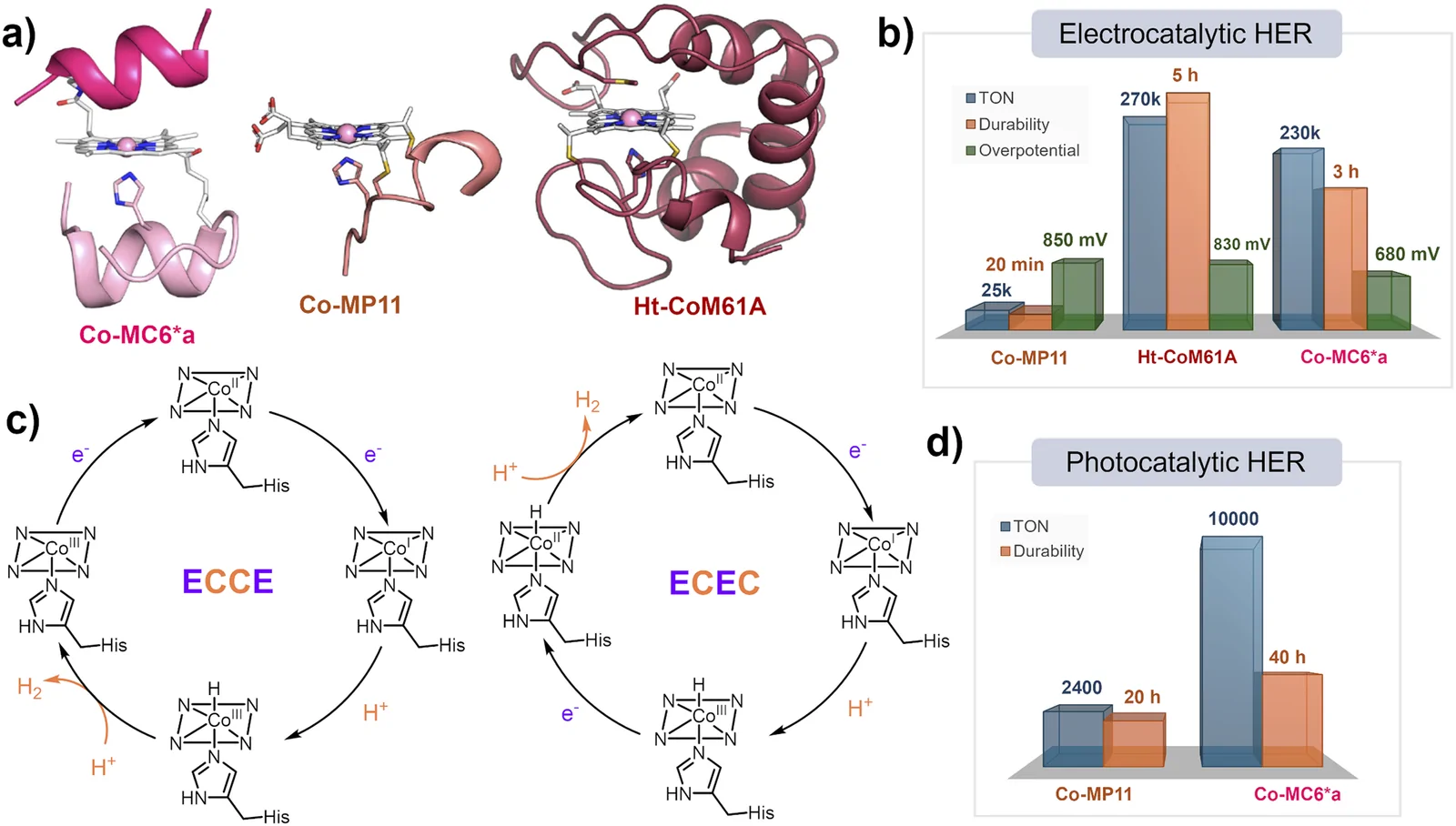
(a) Co-porphyrin containing artificial metalloenzymes for energy-relevant transformations. Reproduced from ref. 130 with permission from MDPI, copyright 2023. (b) Comparative analysis of electrocatalytic Hydrogen Evolution Reaction (HER) parameters among CoMP11, HtCoM61A and CoMC6*a. (c) Catalytic mechanisms for HER, involving both Electrochemical (electron transfer) and Chemical (proton transfer) steps. Adapted from ref. 141 with permission from American Chemical Society, copyright 2020. (d) Comparative analysis of photocatalytic HER parameters among CoMP11 and CoMC6*a.

Inspired by the work of Bren group on Co-porphyrin catalysts derived from cytochrome *c*,^135^ we identified an opportunity to generate a related system by replacing iron with cobalt in MC6*a (Fig. 4a). Accordingly, we joined our efforts with those of the Bren group and investigated the reactivity of CoMC6*a in HER.

In early studies, CoMC6*a was identified as an efficient electrocatalyst for HER in aqueous solution at nearly neutral pH,^81^ reaching an impressive TON of ∼230 000 over 3 h, a high faradaic efficiency (FE = 96–97% after 1 h), and, notably, exhibiting exceptional tolerance to O_2_.^81^ Earlier studies on CoMP11 (cobalt microperoxidase-11)^136^ and Ht-CoM61A (cobalt-substituted *Hydrogenobacter thermophilus* cytochrome *c*552)^137^ offered the opportunity to elucidate the role of the peptide scaffold in influencing overpotential, TON, and catalyst longevity, which are key aspects in electrocatalytic HER (Fig. 4b). CoMP11 is a cobalt-substituted heme–peptide complex derived from cytochrome *c*, bearing a single 11-residue peptide covalently bound to the porphyrin, which provides an axial histidine.^138^ CoMP11 achieved ∼25 000 turnovers at an overpotential of ∼850 mV, but suffered from rapid deactivation, showing significant loss of activity after only ∼20 minutes of electrolysis.^81^ Conversely, Ht-CoM61A is the cobalt-substituted M61A mutant of the thermophilic 80-amino acid protein cytochrome *c*_552_. Its deeply buried active site confers considerably greater robustness than CoMP11, sustaining catalysis for up to 5 h and reaching a TON of ∼270 000.^137^ However, Ht-CoM61A showed a catalytic overpotential of 830 mV, which is nearly unchanged compared to CoMP11. In terms of scaffold complexity, CoMC6*a lays in the middle between the two described systems, featuring an additional distal peptide chain compared to CoMP11, and a smaller size compared to Ht-CoM61A. Its minimal peptide scaffold provides an effective balance between structural complexity and functionality, reaching TON values in the same order of magnitude as Ht-CoM61A, while showing improved longevity and O_2_ tolerance compared to CoMP11. Notably, CoMC6*a exhibits a catalytic overpotential of ∼680 mV at maximum current, outperforming both the simpler and the more complex systems.^81^

These data underline the key role of the peptide scaffold in governing both longevity and efficiency in HER electrocatalysis. Furthermore, cyclic voltammetry experiments conducted in the presence of TFE revealed that the enhanced helical structure in CoMC6*a produces an anodic shift in the catalytic half-wave potential of up to ∼100 mV relative to CoMP11, which conversely lacks an organized secondary structure and shows no significant potential shift under the same conditions. This demonstrates that the scaffold directly modulates the energetic requirements for proton reduction through folding-dependent tuning of the reduction potential. Remarkably, the small differences between CoMC6*a and Ht-CoM61A in terms of TON suggest that the well-designed minimal peptide scaffold of CoMC6*a had a comparable role, in terms of both catalyst robustness and reactivity, with that exerted by the bigger and more structured protein scaffold of Ht-CoM61A.

Building on these results, further studies have focused on elucidating the mechanism of proton reduction promoted by CoMC6*a. Cobalt-catalyzed HER catalysis is generally proposed to proceed through sequences of electron transfer (E) and chemical proton transfer (C) processes, following either an ECEC or an ECCE scheme (Fig. 4c). Central to both pathways is the formation of a reactive Co^I^ species, and its protonation to a transient Co^III^–hydride (Co^III^–H) intermediate.^139^ This latter may be further reduced to a Co^II^–H, and ultimately leads to H_2_ production *via* either a homolytic (bimolecular reaction of two Co^III^–H species) or a heterolytic pathway (protonation of Co^III^–H or Co^II^–H).^132,140^ Previous electrochemical characterization of CoMC6*a in DMF showed quasi-reversible Co^III^/Co^II^ and Co^II^/Co^I^ redox couples, allowing to identify the low-valent Co^I^ species as the catalytically active form,^81^ although in aqueous solution the Co^II^/Co^I^ reduction process is obscured by catalytic currents associated with proton reduction.

A distinctive feature of CoMC6*a is the strong influence of the proton donor on both catalytic behavior and mechanism.^141^ The catalytic potential is governed primarily by the p*K*_a_ of the proton donor rather than its concentration, highlighting that thermodynamics of the process is dictated by proton donor strength. In contrast, the reaction rate is affected by proton accessibility, with steric hindrance slowing catalysis down without altering the underlying thermodynamics. This is consistent with a conformational rearrangement of the distal peptide to facilitate proton delivery, by exposing the distal side of the porphyrin which is otherwise protected by a helix in its folded form.^81,85^ Notably, CoMC6*a exhibits a clear mechanistic switch depending on acidity of the proton donor. Stronger acids enable protonation of both Co^I^ and Co^III^–H intermediates, consistent with an ECCE pathway. On the other hand, weaker acids can only protonate Co^I^ to yield Co^III^–H, requiring an additional reduction step to Co^II^–H before H_2_ is released, consistent with an ECEC pathway (Fig. 4c). This behavior demonstrated that CoMC6*a enables the decoupling of thermodynamic (governed by p*K*_a_) from kinetic (governed by proton accessibility) effects in HER catalysis, providing a valuable platform for tuning reactivity through the properties of the proton source.^141^

Beyond its electrocatalytic activity, CoMC6*a also demonstrated to work under photocatalytic conditions, promoting light-driven HER in aqueous solution.^82^ In the presence of [Ru(bpy)_3_]Cl_2_ (bpy = 2,2′-bipyridine) as a photosensitizer and ascorbate as a sacrificial electron donor, blue light irradiation triggered catalytic HER. This system achieves TON values exceeding 10 000, while maintaining activity over extended periods (up to 40 h). Systematic variation of component concentrations revealed that overall performance is limited primarily by photosensitizer degradation, whereas the catalyst itself remains stable under irradiation. Optimal activity was observed at neutral pH, reflecting the combined influence of ascorbate speciation under acidic conditions and the thermodynamics of Co–H formation at elevated pH values. Further, the retention of activity under mild basic conditions (TON ∼2860 at pH 9.1) suggests a relatively high p*K*_a_ for the key protonation step leading to the Co^III^–H intermediate^82^ compared to those estimated for Co-porphyrins.^142^ Despite its minimal scaffold, CoMC6*a showed the highest longevity among Co-porphyrin-based biomimetic photocatalysts, which included both small-molecule Co-porphyrins (*i.e. meso*-tetrakis(1-methylpyridinium-4-yl)-porphyrin^143^ and *meso*-tetrakis(*p*-sulfonylphenyl)porphyrin)^144^ and Co-porphyrin containing artificial metalloenzymes, including Co-MP11,^145^ Co-αRep,^146^ Co-cyt *b*_562_,^147^ and Co-Mb.^148^ In particular, CoMC6*a outperforms CoMP11 by approximately 10-fold in the TON value and sustains catalysis for longer times (40 h *vs.* 20 h), confirming the trend observed in electrocatalysis while validating again that the distal peptide scaffold plays a key role in protecting the catalyst from degradation during turnover.

**Scheme 4:**
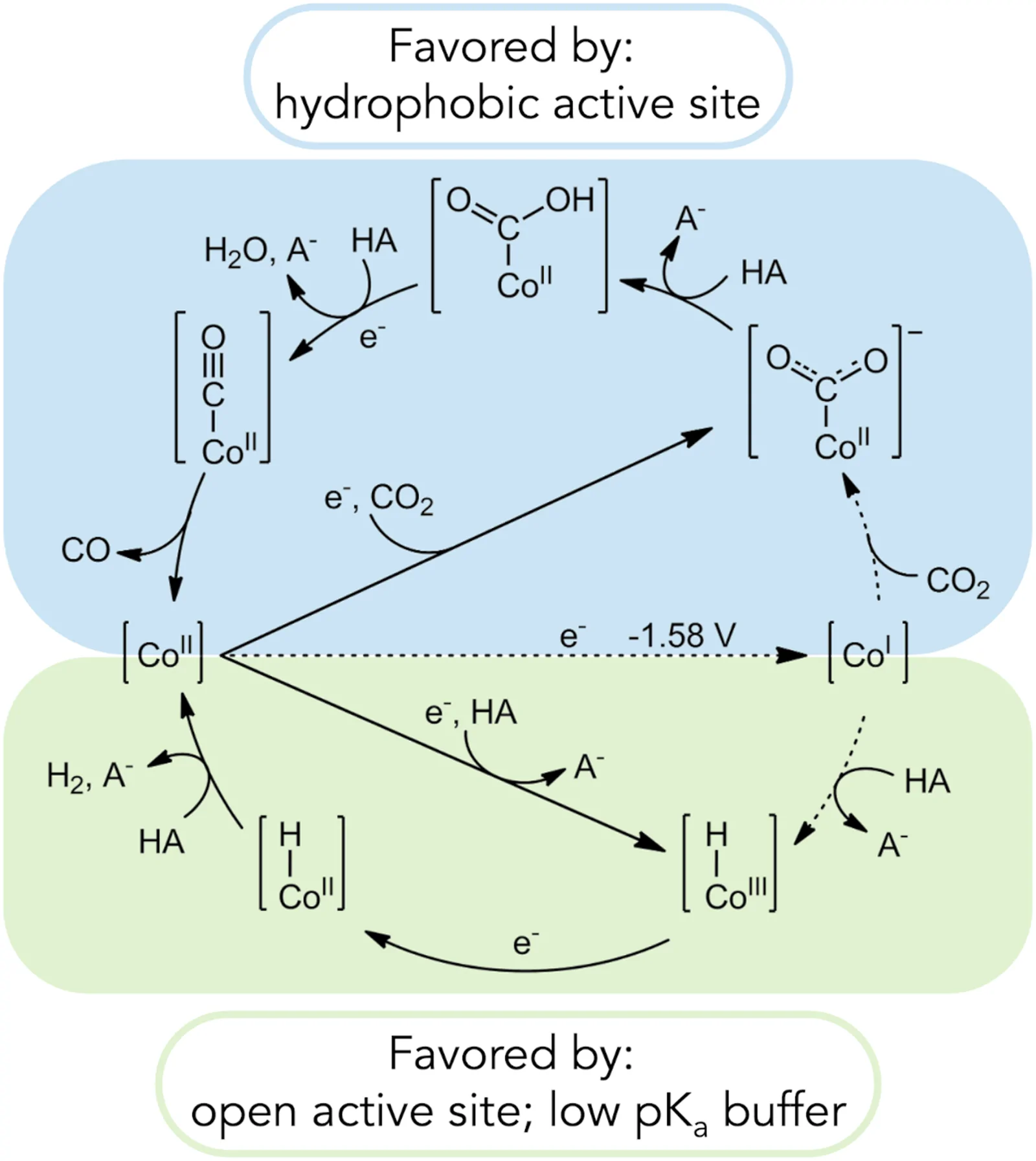
Proposed mechanisms for the competing H_2_ evolution reaction (green box) and CO_2_ reduction reaction (light blue box) catalyzed by CoMC6*a. For clarity, only the cobalt center and the H^+^- or CO_2_-derived species directly coordinated to it are shown. The reduction of Co^II^ to Co^I^ (straight dotted line) occurs at −1.58 V *vs.* Ag/AgCl and is therefore not expected under the experimental conditions employed in the study (−1.2 or −1.4 V *vs.* Ag/AgCl).^85^ Processes that are not observed or expected under these conditions are represented by dotted lines. Adapted from ref. 85 with permission from Royal Society of Chemistry, copyright 2025.

More recently, CoMC6*a has also been investigated for its selectivity between hydrogen evolution and electrocatalytic CO_2_ reduction to CO (CO_2_ + 2H^+^ + 2e^−^ ⇌ CO + H_2_O), an attractive reaction for renewable fuel production.^85^ The catalyst displays tunable selectivity that depends on both the strength (p*K*_a_) of the proton donor and the applied potential. Consistent with the behavior observed for cobalt and iron porphyrins,^149,150^ the use of high-p*K*_a_ buffers (weak proton donors) reduces metal-hydride formation and suppresses H_2_ evolution, thereby enhancing the selectivity for CO_2_ reduction. Product distribution is also strongly influenced by the applied potential. At relatively mild potentials (−1.2 V *vs.* Ag/AgCl), CoMC6*a achieves high selectivity toward CO formation, following a mechanism in which CO_2_ binding is directly coupled to the reduction of Co^II^ (Scheme 4). This enables CO formation without the accumulation of a discrete Co^I^ intermediate and results in high selectivity (FE_CO_ ∼80–88%) that is largely insensitive to the buffer p*K*_a_. The anodic shift of the catalytic onset with increasing CO_2_ pressure further supports a mechanism in which CO_2_ coordination occurs concertedly with electron transfer (Scheme 4). Under CO_2_ at pH ∼6, CoMC6*a exhibits excellent performances across a range of buffers, achieving TON_CO_ ∼14 000 over 24 h with CO : H_2_ ratios of approximately 86 : 5 at −1.2 V *vs.* Ag/AgCl. Remarkably, the catalyst retains substantial activity in the presence of O_2_, a rare feature among molecular CO_2_-reduction systems.^85^

Comparison with CoMP11 again highlights the impact of catalyst structure on robustness and selectivity. While the two catalysts display similarly high selectivity at −1.2 V, CoMC6*a demonstrates significantly greater long-term stability, albeit at the expense of lower initial turnover rates with respect to CoMP11.^85^ This enhanced robustness can be attributed to the distal helix of CoMC6*a, which modulates the local environment of the active site. Importantly, this effect governs selectivity at more negative applied potential (−1.4 V), where the divergence between the two catalysts is most pronounced. The narrow and hydrophobic distal site of CoMC6*a is proposed to limit the access of protonated buffer to the cobalt center, suppressing formation of Co–H intermediates and thus H_2_ evolution, while favoring CO_2_ binding and reduction (Scheme 4). As a result, CoMC6*a maintains high CO selectivity even at more negative potentials, where competing hydrogen evolution becomes dominant in the case of CoMP11.^85^

## Catalysis at the nanoscale: integration of mimochromes into catalytic nanomaterials

**Fig. 5:**
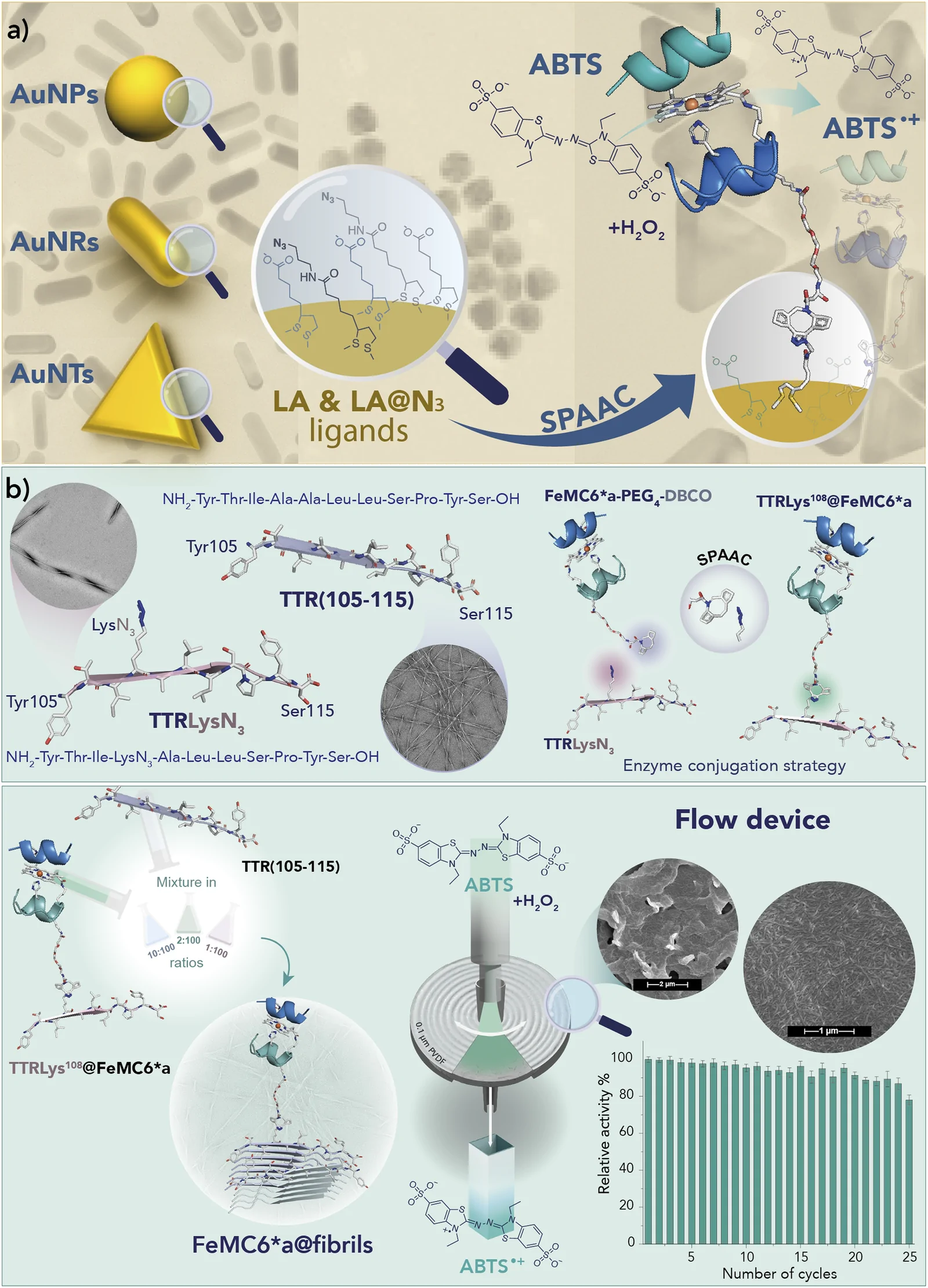
Catalytic nanosized materials obtained through the conjugation of FeMC6*a to gold nanomaterials and amyloid fibrils. Panel (a) depicts differently-shaped gold nanoparticles (AuNPs), nanorods (AuNRs) and triangular nanoprisms (AuNTs) functionalized with a lipoic acid (LA) and its azide-derivative (LA@N_3_) for click conjugation of FeMC6*a-PEG_4_-DBCO. The resulting nanomaterials were evaluated for catalytic activity using ABTS as a model substrate. Adapted from ref. 152 with permission from Royal Society of Chemistry, copyright 2024. Panel (b) depicts the amino acid sequences of the TTR(105–115) peptide and its modified analogue TTRLysN_3_, together with a schematic representation of the functionalization of TTRLysN_3_ with FeMC6*a, leading to the formation of the TTRLys^108^@FeMC6*a conjugate. The assembly of TTRLys^108^@FeMC6*a with TTR(105–115) to form catalytic fibrils and the flow device adopted to perform multiple cycles of ABTS oxidation are also shown. Adapted from ref. 41 with permission from John Wiley and Sons, copyright 2026.

The incorporation of peroxidase-like catalysts into nanostructured materials is an effective strategy to bridge homogeneous and heterogeneous catalysis, enabling the design of functional systems with enhanced stability, recyclability, and tunable activity. The compact size, robustness and catalytic efficiency of the MC scaffold make it a uniquely suited candidate, providing practical advantages that bulkier natural enzymes rarely provide. Early efforts focused on the immobilization of FeMC6*a onto azide-functionalized spherical gold nanoparticles *via* strain-promoted azide–alkyne cycloaddition click chemistry (SPAAC), after functionalization of FeMC6*a with a PEG_4_-dibenzocyclooctyne (DBCO) linker, yielding stable bioconjugates that retain both structural integrity and a significant fraction of catalytic activity (Fig. 5a).^151^ This site-specific nature of the immobilization strategy ensures strong and selective anchoring of the catalyst while minimally perturbing the active site, unlike large natural enzymes, which often suffer from partial denaturation or loss of activity upon immobilization.

Subsequent studies have expanded the scope of FeMC6*a-based nanomaterials by exploring the effect of nanoparticle architecture on catalytic performance. The conjugation of FeMC6*a to anisotropic gold nanostructures, such as nanorods and triangular nanoprisms (Fig. 5a), has revealed that the shape of the nanomaterial critically influences its catalytic behavior.^152^ Differences in surface curvature, edge structure, and local environment modulate enzyme orientation and substrate accessibility, demonstrating that nanoscale features of the support can be used as active design parameters to tune reactivity. Importantly, the small size of FeMC6*a allows for high surface loading without significantly inducing crowding effects, typically reducing catalysis at the nanomaterial interface.^152^ The diagnostic relevance of miniaturization becomes even more evident when considering applications in biosensing and signal amplification. The integration of FeMC6*a in lateral flow immunoassays represents a striking example of how a small, robust peroxidase mimic can outperform traditional enzymatic labels in terms of stability and operational simplicity.^153^ When conjugated to nanoparticles used as reporters in immunoassays, FeMC6*a enables efficient catalytic signal amplification while maintaining compatibility with the constraints imposed by the assay. Its reduced size facilitates higher loading on the nanoparticle surface and improves accessibility to substrates, ultimately leading to enhanced sensitivity. At the same time, the synthetic nature of the catalyst ensures batch-to-batch reproducibility and resistance to environmental stress factors, addressing key limitations associated with natural enzymes such as HRP.

A complementary approach to inorganic nanostructures employs self-assembling peptide architectures, such as amyloid fibrils, which offer highly ordered, robust, and chemically versatile platforms for enzyme immobilization.^41^ The covalent conjugation of FeMC6*a to amyloidogenic peptides derived from human transthyretin, TTR(105–115), has enabled the formation of catalytic fibrils in which the enzyme is distributed along the nanostructure (Fig. 5b).^154^ In these systems, the degree of functionalization is finely tuned by controlling the ratio between modified and unmodified peptide monomers, and an optimal enzyme loading has been identified, at which the immobilized catalyst exceeds the activity of the freely diffusing enzyme. The exceptional stability of amyloid fibrils translates into nanomaterials that exhibit improved durability and reusability, as demonstrated by their successful integration into a flow-reactor system for multiple catalytic cycles (Fig. 5b).

Overall, these studies show that miniaturization of peroxidases is not merely an exercise of structural simplification, but a practical strategy to design functional nanomaterials. The reduced size and high tolerance to non-physiological conditions by FeMC6*a allow a high level of control over loading, orientation and functionalization, that is difficult to achieve with natural enzymes. These features open up concrete applications of miniaturized enzymes in biosensing and heterogeneous catalysis.

## Future outlook

This perspective summarizes more than two decades of research on miniaturized heme enzymes, leading to compounds with highly effective, versatile, and tunable reactivity. The evolution of the MC scaffold, from the earliest prototype MC1 up to the most effective member MC6*a, demonstrates that miniaturization is a valid alternative to the existing approaches in the design and engineering of artificial heme enzymes. By retaining the minimal structural elements required for catalytic activity, this strategy has produced catalysts that match, and in many cases surpass, the performances of natural or artificial metalloenzymes of far higher complexity, without the need for elaborate protein environments or extensive engineering techniques. The simplicity of the MC scaffold has been identified as a crucial feature to its broad substrate promiscuity, allowing the catalyst to process structurally diverse molecules in oxidative chemistry. Further, replacement of iron center with other transition metals has opened access to reactivities beyond peroxidase.

Looking ahead, a key challenge for the next generation of mimochromes will be the rational design of substrate-binding interactions within the minimal scaffold. This will likely require moving beyond the current “swiss-knife” nature of MC6*a. While its inherent promiscuity is a major advantage for broad-scope catalysis, it may become a limitation when high stereo- or enantioselectivity is required. Engineering a well-defined substrate-binding pocket within such a compact peptide scaffold is an ambitious goal, as it will likely require an increase in the structural complexity of MCs, while preserving their minimalist design. In this context, the straightforward synthesis of the MC scaffold should allow rapid preparation of tailored analogues for specific substrates and transformations.

Further, one of the most compelling directions is the development of new mimochrome analogues inspired by unspecific peroxygenases (UPOs), which are capable of selectively oxidizing C–H bonds with dissociation energies approaching or exceeding 100 kcal mol^−1^. Achieving this level of reactivity within a miniaturized scaffold would mark a major step forward, positioning mimochromes as effective tools for late-stage functionalization of complex molecules. In this context, the remarkable tolerance of the MC scaffold toward organic solvents, combined with its synthetic accessibility and stability, provide a concrete advantage for applications in the synthesis of fine chemicals and waste valorization, where catalytic upgrading of low-value compounds into more complex and valuable products is increasingly desirable. Beyond oxidative chemistry, the study of abiological transformations, so far achieved only with complex protein scaffolds, represents a logical extension and an open challenge for the MC scaffold.

In parallel, the integration of mimochromes into nanostructured materials is an increasingly important area of development. Besides the reported proof-of-concept diagnostic device, the full potential of these miniaturized enzymes is yet to be explored. Future efforts are expected to lead to the construction of multifunctional platforms in which mimochromes are coupled with other enzymes, redox mediators, or photoactive components to enable cascade and light-driven reactions. In particular, photochemical activation strategies could expand the scope of reactivity beyond energy-related processes such as H_2_ evolution and CO_2_ reduction, including hydrogen atom transfer reactions or carbene-transfer transformations.

As the demand for more sustainable chemical technologies continues to grow, future research will also focus on the development of greener and more scalable synthetic methodologies for MC production, minimizing the environmental impact while preserving the simplicity that make these systems unique. Altogether, the continued evolution of mimochromes will establish miniaturized artificial metalloenzymes not merely as simplified models of natural biocatalysts, but as a distinct and versatile class of catalysts with tangible applications in synthetic chemistry, environmental remediation, diagnostics, and renewable energy production.

## Author contributions

Linda Leone: conceptualization, visualization, writing – original draft, writing – review and editing; Daniele D'Alonzo: visualization, writing – original draft, writing – review and editing; Roberta Russo: visualization, writing – original draft; Alessandra Esposito: writing – review and editing; Angela Lombardi: conceptualization, funding acquisition, writing – review and editing.

## Conflicts of interest

There are no conflicts of interest to declare.

## Data Availability

No primary research results, software or code have been included and no new data were generated or analysed as part of this review.
